# Supplementary material for: Repeat associated mechanisms of genome evolution and function revealed by the Mus caroli and Mus pahari genomes
Source: Genome Res. 2018 Apr;28(4):448–59. doi: 10.1101/gr.234096.117 (PMC5880236; doi:10.1101/gr.234096.117)
Supplement: Supplemental Material [file supp_28_4_448__index.html]

Repeat associated mechanisms of genome evolution and function revealed by the Mus caroli and Mus pahari genomes — Repeat associated mechanisms of genome evolution and function revealed by the Mus caroli and Mus pahari genomes — Supplemental Material 

# Repeat associated mechanisms of genome evolution and function revealed by the *Mus caroli* and *Mus pahari* genomes

## Supplemental Material

- Supplemental\_Figures.pdf
- Supplemental\_Methods.pdf
