# Supplementary material for: Repeat associated mechanisms of genome evolution and function revealed by the Mus caroli and Mus pahari genomes
Source: Genome Res. 2018 Apr;28(4):448–59. doi: 10.1101/gr.234096.117 (PMC5880236; doi:10.1101/gr.234096.117)
Supplement: Supplemental Material [file supp_gr.234096.117_Supplemental_Figures.pdf]

Supplemental Figures

Repeat associated mechanisms of genome evolution and function revealed by *the Mus caroli* and *Mus pahari* genomes

David Thybert[1,2], Maša Roller[1], Fábio C.P. Navarro[3], Ian Fiddes[4], Ian Streeter[1], Christine Feig[5], David Martin-Galvez[1], Mikhail Kolmogorov[6], Václav Janoušek[7], Wasiu Akanni[1], Bronwen Aken[1], Sarah Aldridge[5,8], Varshith Chakrapani[1], William Chow[8], Laura Clarke[1], Carla Cummins[1], Anthony Doran[8], Matthew Dunn[8], Leo Goodstadt[9], Kerstin Howe[3], Matthew Howell[1], Ambre-Aurore Josselin[1], Robert C. Karn[10], Christina M. Laukaitis[10], Lilue Jingtao[8], Fergal Martin[1], Matthieu Muffato[1], Stefanie Nachtweide[11], Michael A. Quail[8], Cristina Sisu[3], Mario Stanke[11], Klara Stefflova[5], Cock Van Oosterhout[12], Frederic Veyrunes[13], Ben Ward[2], Fengtang Yang[8], Golbahar Yazdanifar[10], Amonida Zadissa[1], David J. Adams[8], Alvis Brazma[1], Mark Gerstein[3], Benedict Paten[4], Son Pham[14], Thomas M. Keane[1,8], Duncan T Odom[5,8]*, Paul Flicek[1,8]*

[1] European Molecular Biology Laboratory, European Bioinformatics Institute, Wellcome Genome Campus, Hinxton, Cambridge, CB10 1SD, United Kingdom
[2] Earlham Institute, Norwich research Park, Norwich, NR4 7UH, United Kingdom
[3] Yale University Medical School, Computational Biology and Bioinformatics Program, New Haven, Connecticut 06520, USA
[4] Department of Biomolecular Engineering, University of California, Santa Cruz, California 95064, USA
[5] University of Cambridge, Cancer Research UK Cambridge Institute, Robinson Way, Cambridge CB2 0RE, UK
[6] Department of Computer Science and Engineering, University of California, San Diego, La Jolla, CA 92092
[7] Department of Zoology, Faculty of Science, Charles University in Prague, Prague, Czech Republic Institute of Vertebrate Biology, ASCR, Brno, Czech Republic
[8] Wellcome Sanger Institute, Wellcome Genome Campus, Hinxton, Cambridge, CB10 1SA, United Kingdom
[9] Wellcome Trust Centre for Human Genetics, Oxford, UK.
[10] Department of Medicine, College of Medicine, University of Arizona.
[11] Institute of Mathematics and Computer Science, University of Greifswald, Greifswald, 17487, Germany
[12] School of Environmental Sciences, University of East Anglia, Norwich Research Park, Norwich, United Kingdom
[13] Institut des Sciences de l'Evolution de Montpellier, Université Montpellier / CNRS, 34095 Montpellier, France
[14] Bioturing Inc, San Diego, California

Corresponding authors: DTO (duncan.odom@cruk.cam.ac.uk); PF (flicek@ebi.ac.uk)

A

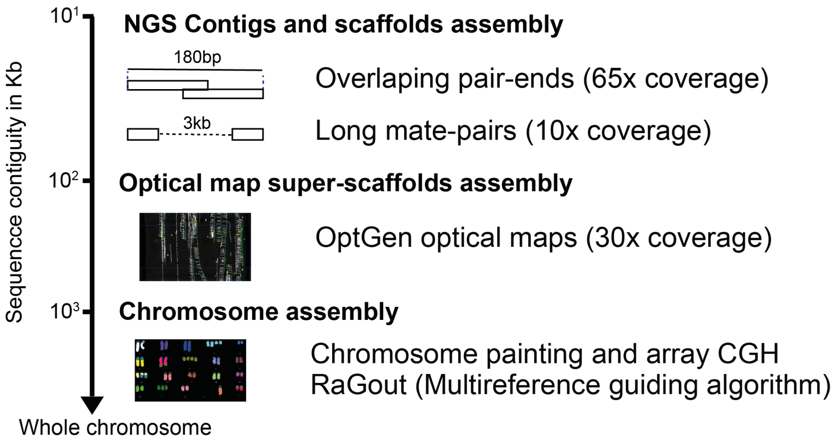

B

**Species**	N50 contigs (Kb)	N50 scaffolds (Mb)	Total Size (Mb)	Size chromosome (Mb)	Size unassembled (Mb)	Fraction gaps (%)	Variation with reference(%)
Mus musculus	24.8	16.9	2,639	2,633	5	2.82	-
Mus caroli	**25.9**	**4.3**	**2,556**	**2,499**	**57**	**10.96**	**3.13 ***
Mus pahari	**24.4**	**3.6**	**2,477**	**2,424**	**53**	**9.48**	**6.13 ***
Rattus norvegic	38.0	5.4	2,866	2,778	88	4.89	8.62 *
Hommo sapiens	21.7	0.3	3,042	3,031	11	3.95	-
Pan troglodytes	15.7	8.6	3,283	3,120	163	11.73	7.91 **
Gorrilla gorilla	11.8	0.9	3,040	2,917	123	10.78	0.06 **
Pongo abelii	15.5	0.7	3,371	3,029	342	10.22	10.8 **

C

**Species**	**Variant**	**Indels**	**SNV**	**Error every Kb**	**Frequency**
Mus caroli	121,159	22,146	99,013	25.24	$3.96\ 10^{-05}$
Mus pahari	133,364	52,702	80,662	30.71	$3.25\ 10^{-05}$

D

	**Insertion**		**Deletion**		**Total**	
Species	No regions	Fraction %	No regions	Fraction %	No regions	Fraction %
Mus caroli	1,086	1.84	1949	1.25	3035	3.1
Mus pahari	723	1.76	968	0.78	1691	2.5

Supplemental Figure S1. Genome sequencing and assembly strategy to create an optimal assembly of chromosomes at different contiguity level.

A. Description of the strategy for sequencing and assembly of the *Mus caroli* and *Mus pahari* genomes

B. Assembly statistics of the four Muridae and Hominidae genomes. The genomes assembled in this study are in bold. The N50 statistics for the genome not assembled in this study come from their initial genome paper.

C. Nucleotide error rates estimated by calling single nucleotide variants from mate-pair libraries mapped to the corresponding final assembly (**Supplemental Methods SM1.14**).

D. Estimated assembly error rates from identified inconsistencies between the corresponding optical maps aligned to the final assembly. Insertion is defined as a fragment present in the final assembly and not found in the optical map data. Deletion is defined as a fragment found in the optical map data and not in the final assembly.

A

Species	Protein coding	Non coding RNA	To be experimentally confirmed	Total
Mus musculus	22,501	12,923	2,264	37,688
Mus caroli	20,323	10,069	1,978	32,370
Mus pahari	20,029	9,336	1,847	31,212
Rattus norvegicus	22,277	8,825	25	31,127
Human	20,246	24,690	1,053	45,989
Chimpanzee	18,759	8,681	0	27,440
Gorilla	20,962	6,701	0	27,663
Orangutan	20,424	6,996	0	27,420

B

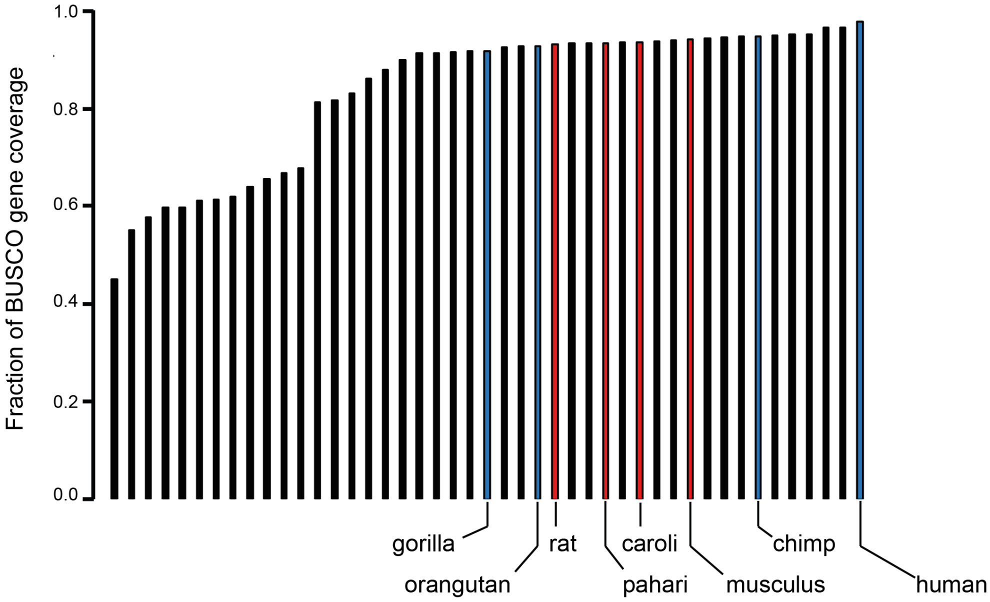

Supplemental Figure S2. *Mus caroli* and *Mus pahari* shows similar annotation statistics and gene completeness than the primates genome sequenced with equivalent technology.

A. Annotation statistics for the four Muridae and Hominidae genomes. *Mus caroli* and *Mus pahari* have been annotated by integrating three different annotation pipelines and RNA-seq data (**Supplemental Methods SM1.7**). The annotation of the other genomes is from Ensembl v83.

B. Gene completeness as measured by the BUSCO dataset for all mammalian genomes available in Ensembl v83 (only the four Muridae (red) and four Hominidae (blue) are labeled). The plot represents the median of the fraction aligned for those members of the BUSCO gene set that align to the genome. The Muridae and Hominidae genomes have similar completeness.

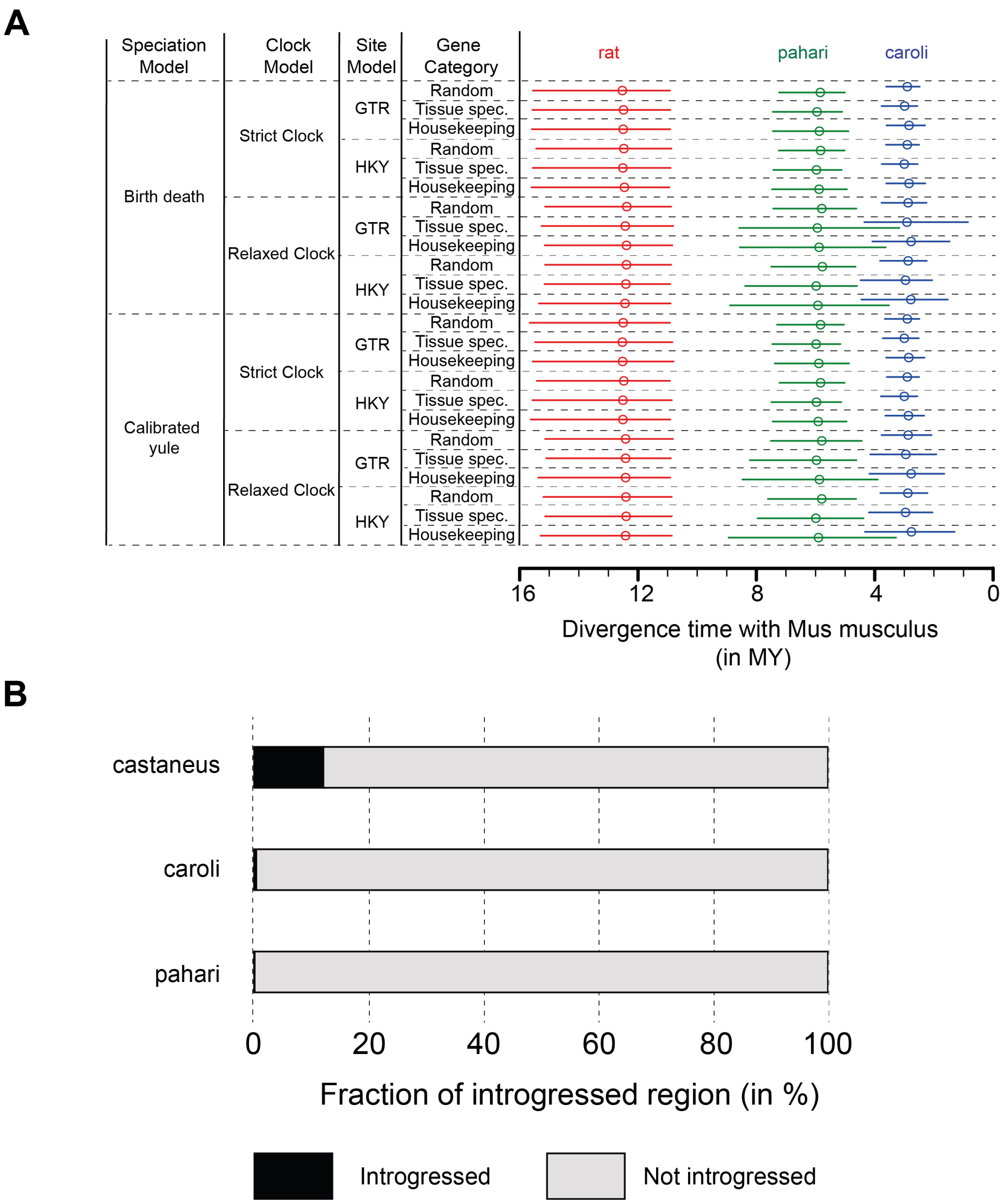

Supplemental Figure S3. Divergence time estimation for *Mus musculus*, *Mus caroli* and *Mus pahari* is robust to the model used.

A. Estimation of *Mus caroli, Mus pahari* and Rat divergence time using different evolutionary models and different sets of coding genes.

B. Fraction of the genome introgressed with *Mus musculus*. *Mus castaneus* is used as positive control.

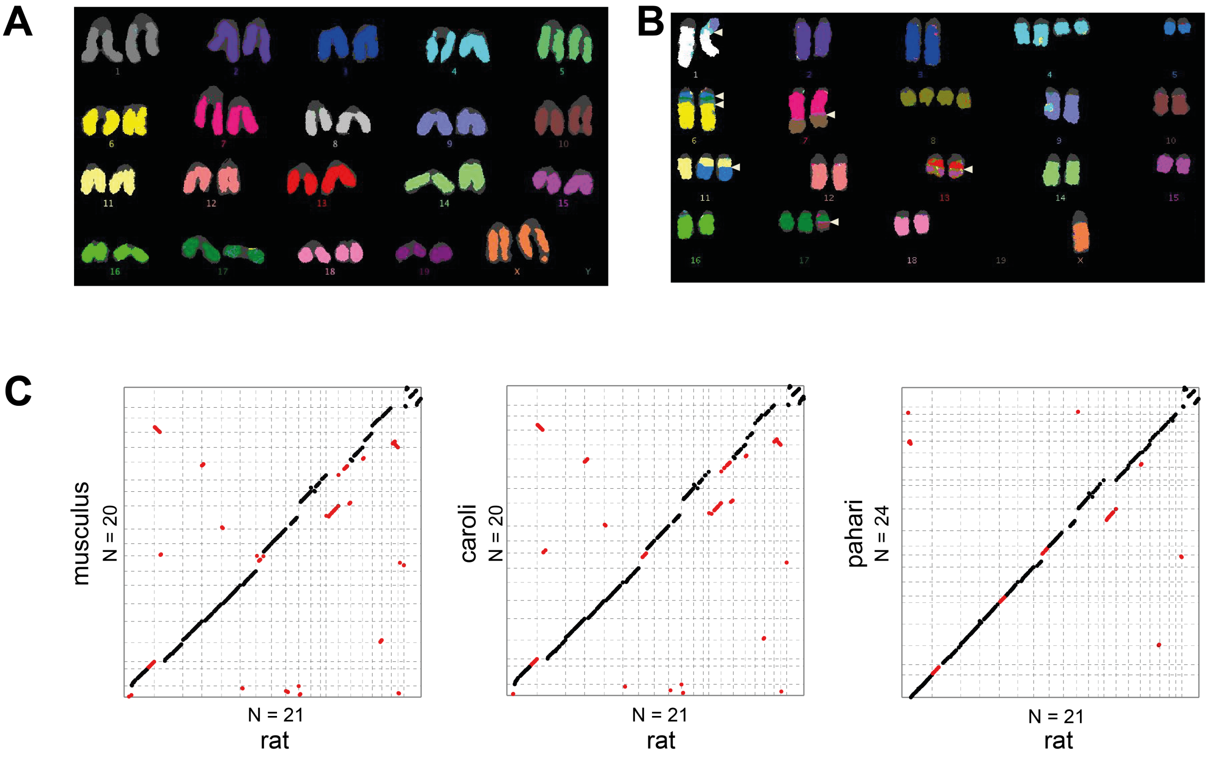

Supplemental Figure S4. The ancestor of *Mus musculus* and *Mus caroli* underwent a largescale rearrangement as compared with *Mus pahari*.

A. DNA FISH of *Mus caroli* karyotype using *Mus musculus* probs.

B. DNA FISH of *Mus pahari* karyotype using *Mus musculus* probes. White arrows identify the break points of major rearrangements.

C. Dot plot showing pairwise comparison of the rat chromosomes with the three *Mus* species. The inter-chromosomal rearrangements are shown in red.

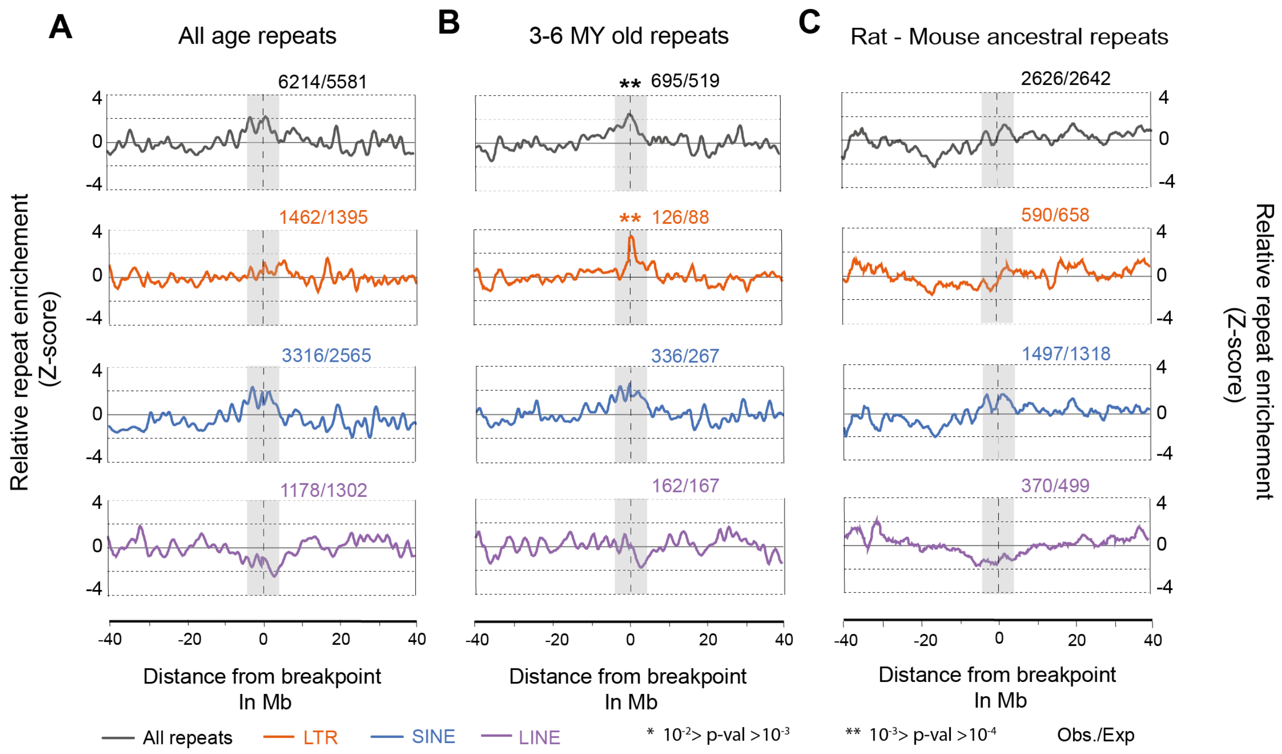

Supplemental Figure S5. The inter-chromosomal break points between *Mus musculus* and *Mus pahari* are enriched with 3-6MY old LTR retrotransposons.

A. Density of all retrotransposons regardless of age plotted by distance to break points of inter-chromosomal rearrangements larger than 3MB. No significant enrichment was observed.

B. Density of 3-6MY old retrotransposons plotted by distance to break points of inter-chromosomal rearrangements larger than 3MB. These retrotransposons appeared concurrent to the punctate event of chromosomal rearrangement. LTRs are significantly enriched at these break points.

C. Density of the mouse-rat ancestral repeats by distance to break points of inter-chromosomal rearrangements larger than 3MB. No significant enrichment was observed.

For A-C, the ratio on the top of each plot show the number of observed element relative to the expected (**Supplemental Methods SM2.3**).

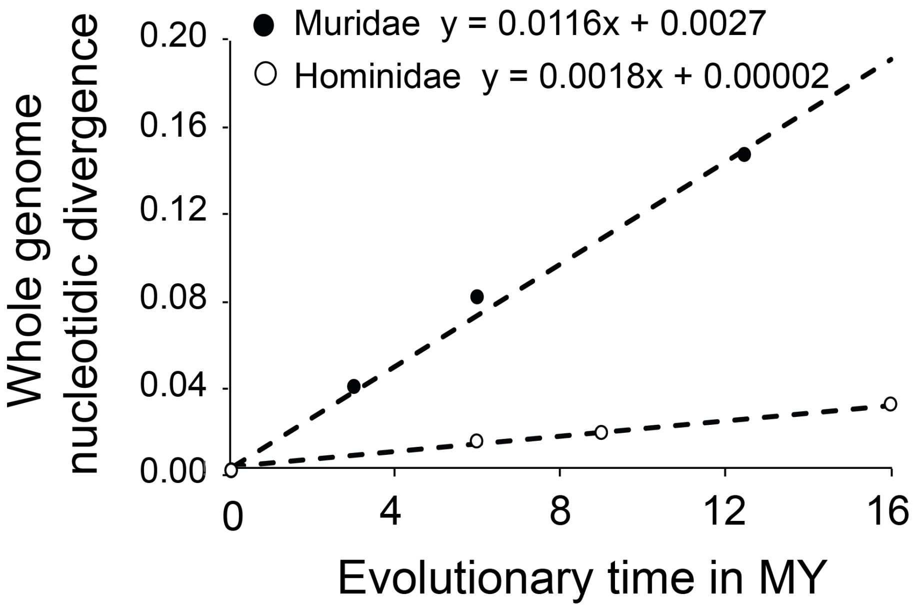

Supplemental Figure S6. Acceleration of Whole genome nucleotide mutational rates in the Muridae lineage. Nucleotide evolutionary rate calculated from whole genome in Muridae (black) and in Hominidae (white).

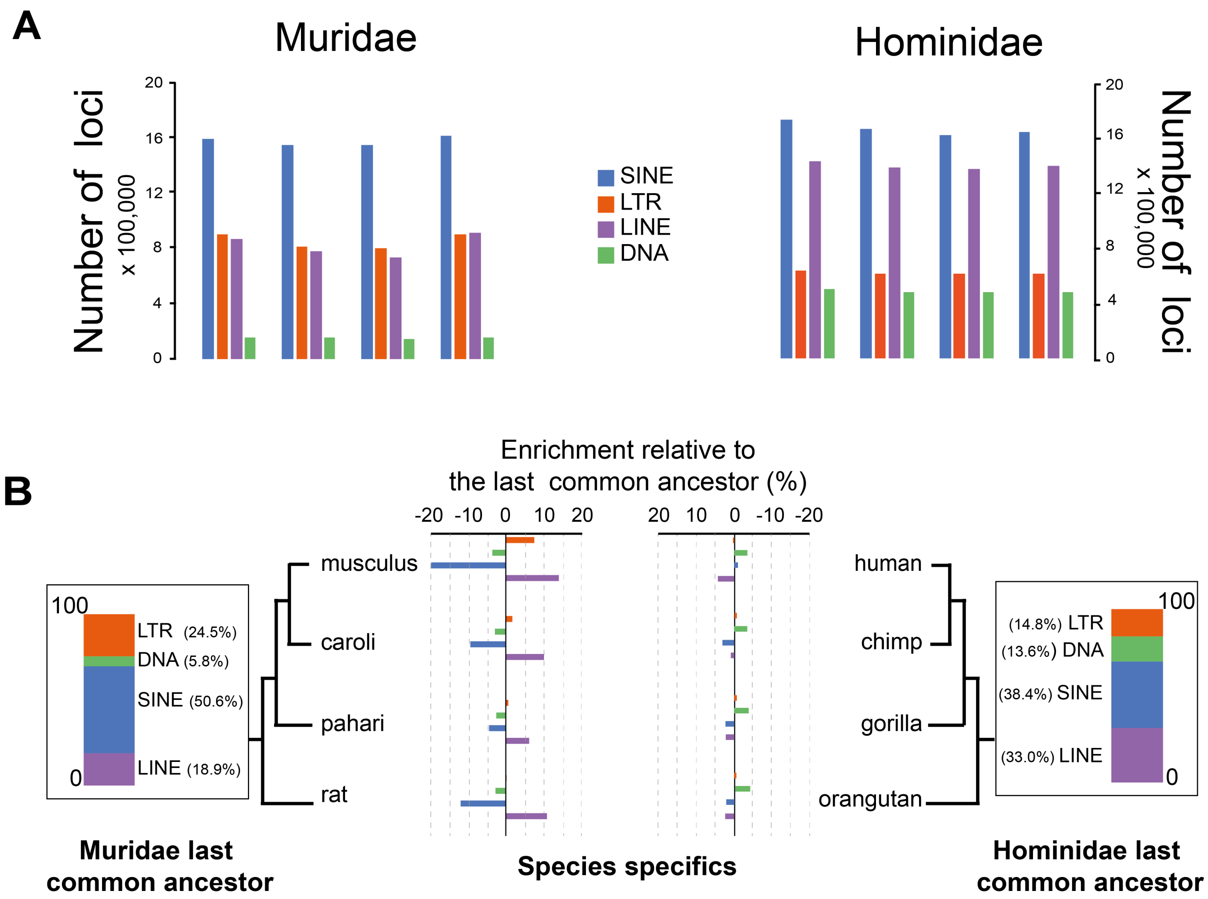

Supplemental Figure S7. Transposable elements have different activity history between the two lineages.

A. Breakdown of the number of transposable elements for each class in the species of the two lineages. The numbers of transposable elements from the four major classes are highly stable intra-lineage and vary between lineages. LINE elements tend to be shorter in Hominidae compared to Muridae (average 382 bp vs 563 bp) meaning that even though the number of loci differ between the clades, the genome coverage is approximately the same (e.g. mouse 19.1%; human 18.7%).

B. Species-specific transposable elements are enriched in LINEs in Muridae. The enrichment has been calculated with regard to a set of ancestral transposable elements. Statistical significance uses Fisher's exact test.

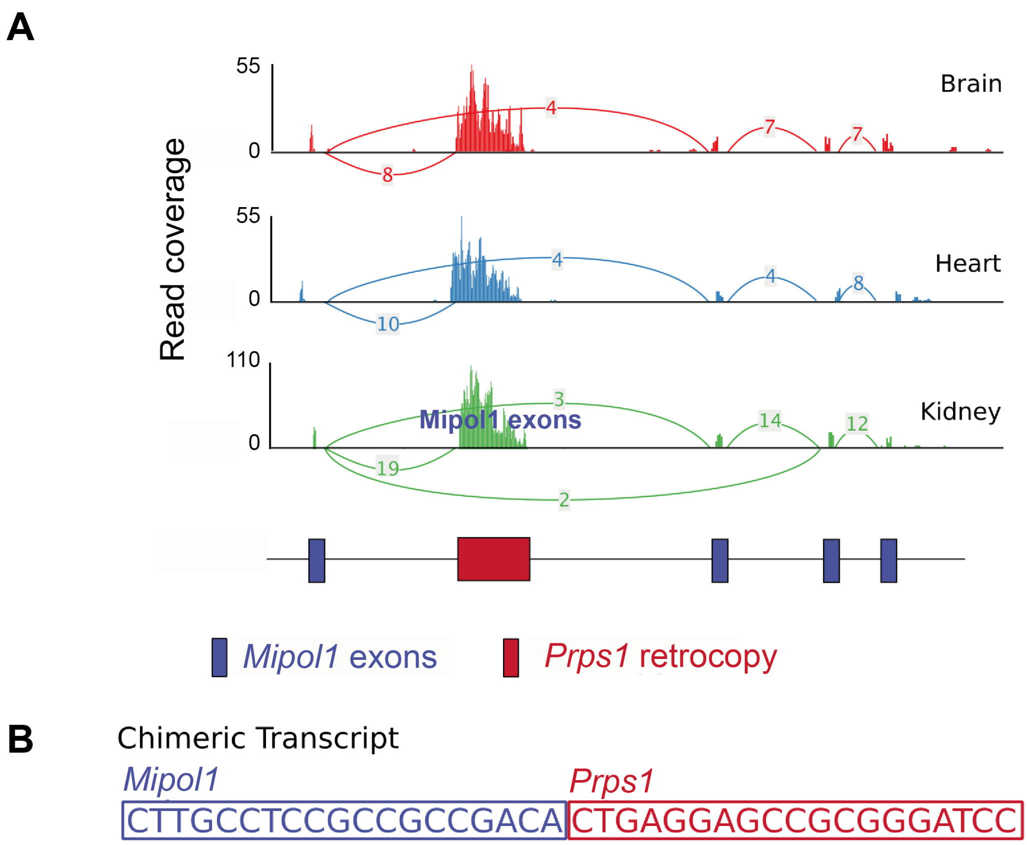

Supplemental Figure S8. Example of a genomic locus carrying a chimeric gene that involves the host gene *Mipol1* and a *Prps1* retrocopy.

A Gene expression profile of the different exons of the chimeric gene in the brain, heart and kidney. The colored arcs represent links between exons in the spliced transcripts. The label on each arc represents the number of reads supporting the association between exons in the final spliced transcript.

B Nucleotide sequence of the chimeric transcript around the *Mipol1*-*Prps1* junction

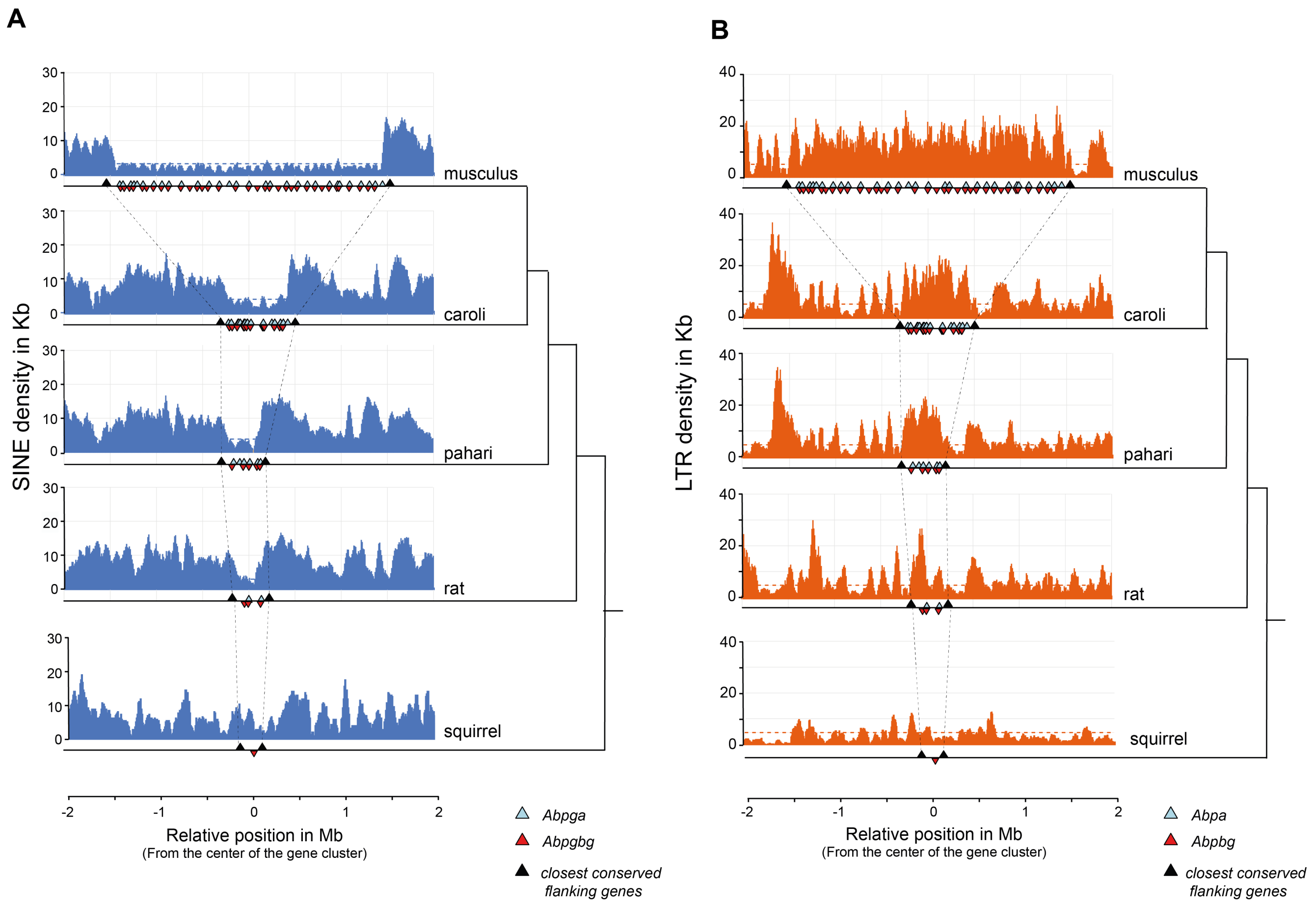

Figure S9. LTR retrotransposons are enriched in the *Shbg* gene cluster while SINEs are depleted.

A Density of SINE elements in the *Abp* gene cluster of the *Mus musculus*, *Mus caroli*, *Mus pahari*, the rat genomes and the squirrel. The blue and red triangles represent the *Abp* genes (*Abpa* in blue, *Abpbg* in red); black triangles represent the closest flanking genes (upstream: *Scn1b* and downstream : *Gpi1*) shared by the four Muridae species.

B Density of LTR elements in the *Abp* gene cluster of the *Mus musculus*, *Mus caroli*, *Mus pahari,*the rat genomes and the squirrel. The blue and red triangles represent the *Abp* genes (*Abpa* in blue, *Abpbg* in red); the black triangles represent the closest flanking genes (upstream: *Scn1b* and downstream : *Gpi1*) shared by the four Muridae species.

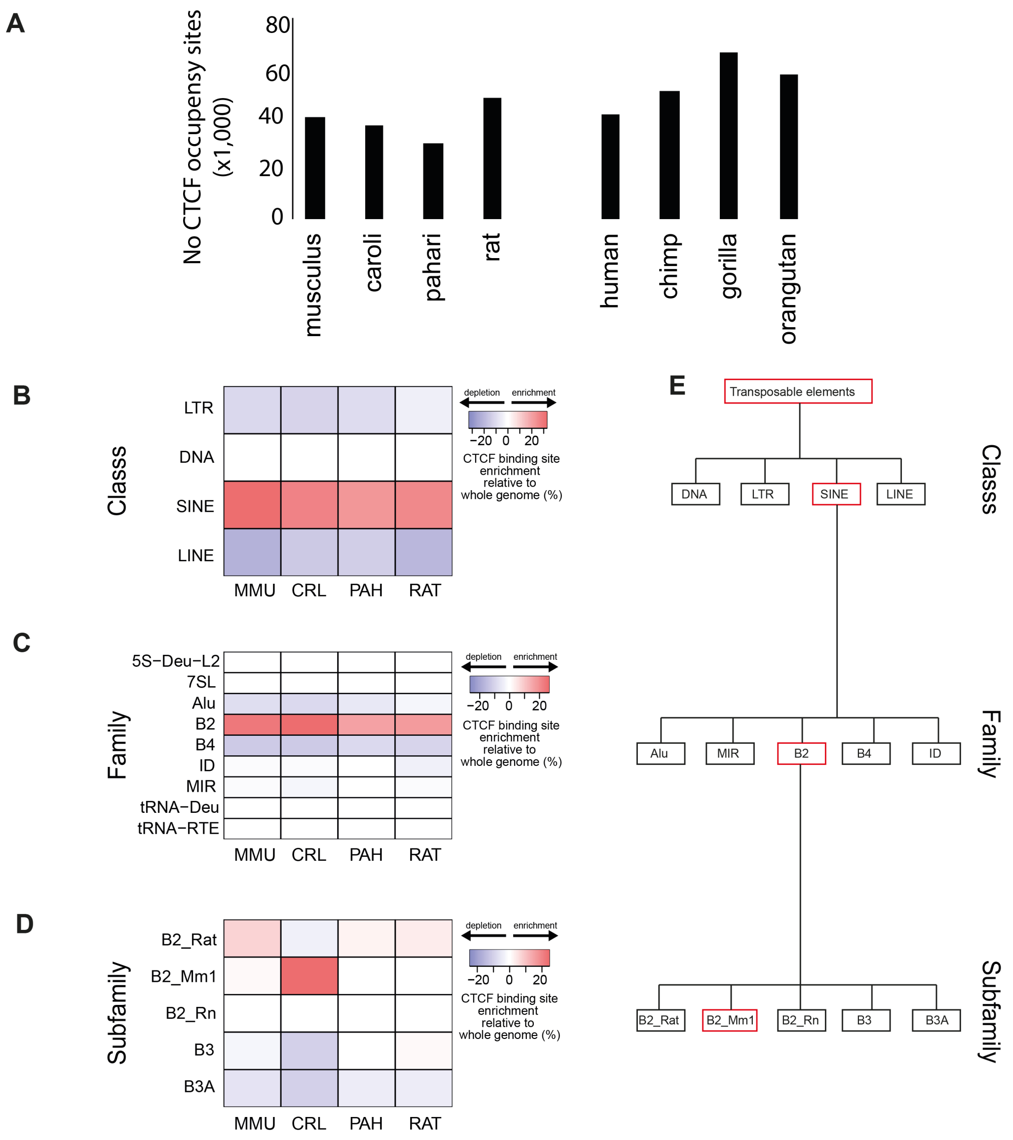

Supplemental Figure S10. Mus caroli show a species-specific enrichment of SINE B2_Mm1 elements carrying CTCF binding.

A. Breakdown of the number CTCF binding sites identified in the Muridae and Hominidae species

B. Overrepresentation of the class of the transposable elements carrying CTCF binding sites

C. Overrepresentation of the family of the SINE elements carrying CTCF binding sites

D. Overrepresentation of the sub-family of the B2 elements carrying CTCF binding sites.

In B-D MMU= *Mus musculus*; CRL= *Mus caroli*; PAH= *Mus pahari*; RAT= Rat

E. Classification hierarchies of the SINE B2 subfamilies**.** The red boxes show the classification hierarchy of the B2_mm1 elements.

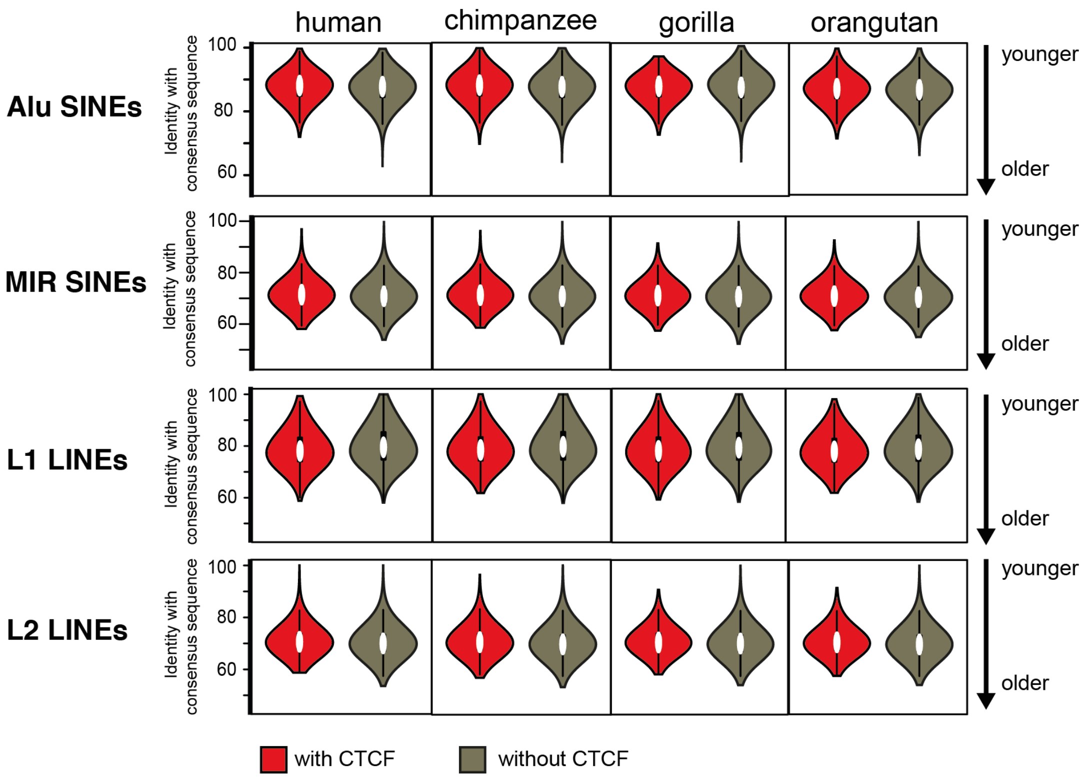

Supplemental Figure S11. Identity plot of each transposable element with its subfamily consensus in the four Hominidae species. Elements carrying CTCF are in red and those not carrying CTCF are in brown.

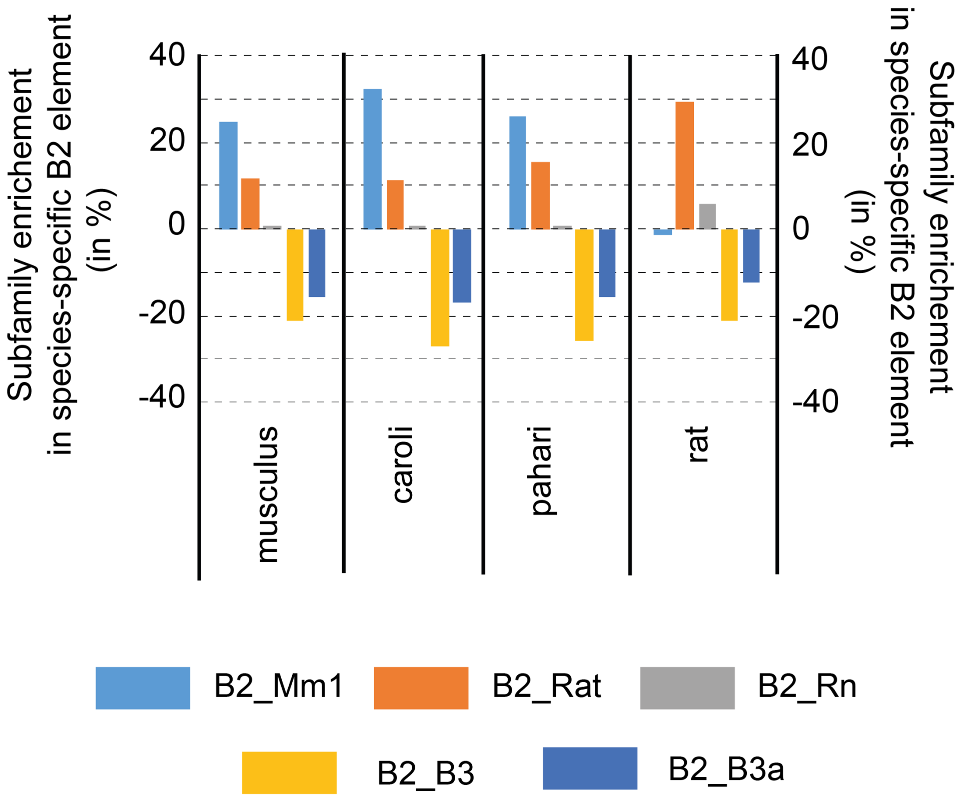

Supplemental Figure S12. The species-specific SINE B2_Mm1 repeats are enriched in the three *Mus* species. SINE B2_Mm1 repeats have a species-specific enrichment limited to the three *Mus* species when compared to a set of ancestral transposable elements.

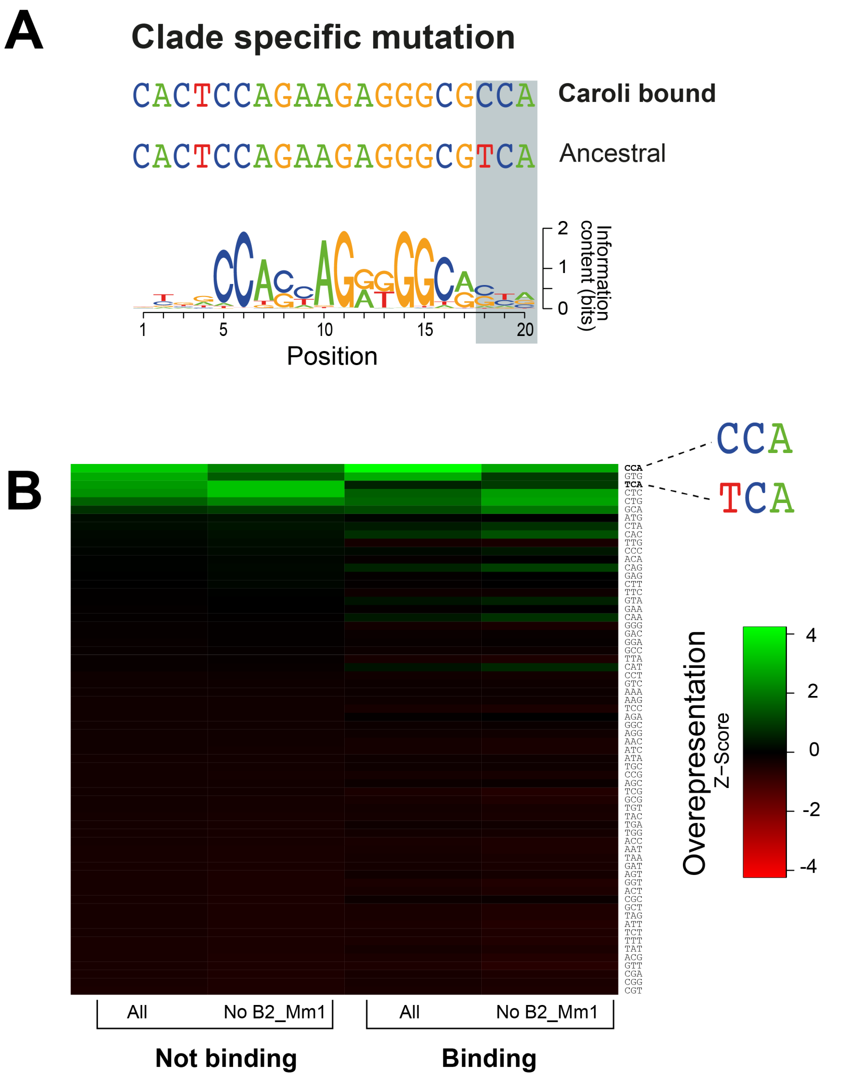

Supplemental Figure S13. Over and under representation of specific trinucleotides in bound and unbound CTCF motifs.

A. Clade-specific trinucleotide CCA and the ancestral trinucleotide TCA from positions 18 to 20 in the CTCF binding motif.

B. Occurrence of all possible trinucleotides in positions 18 to 20 in the CTCF motif bound and not bound by CTCF. The color gradient indicates the level of over/under-representation of each trinucleotide based on a z-score of the number of occurrences (**Supplemental Methods SM5.8**).

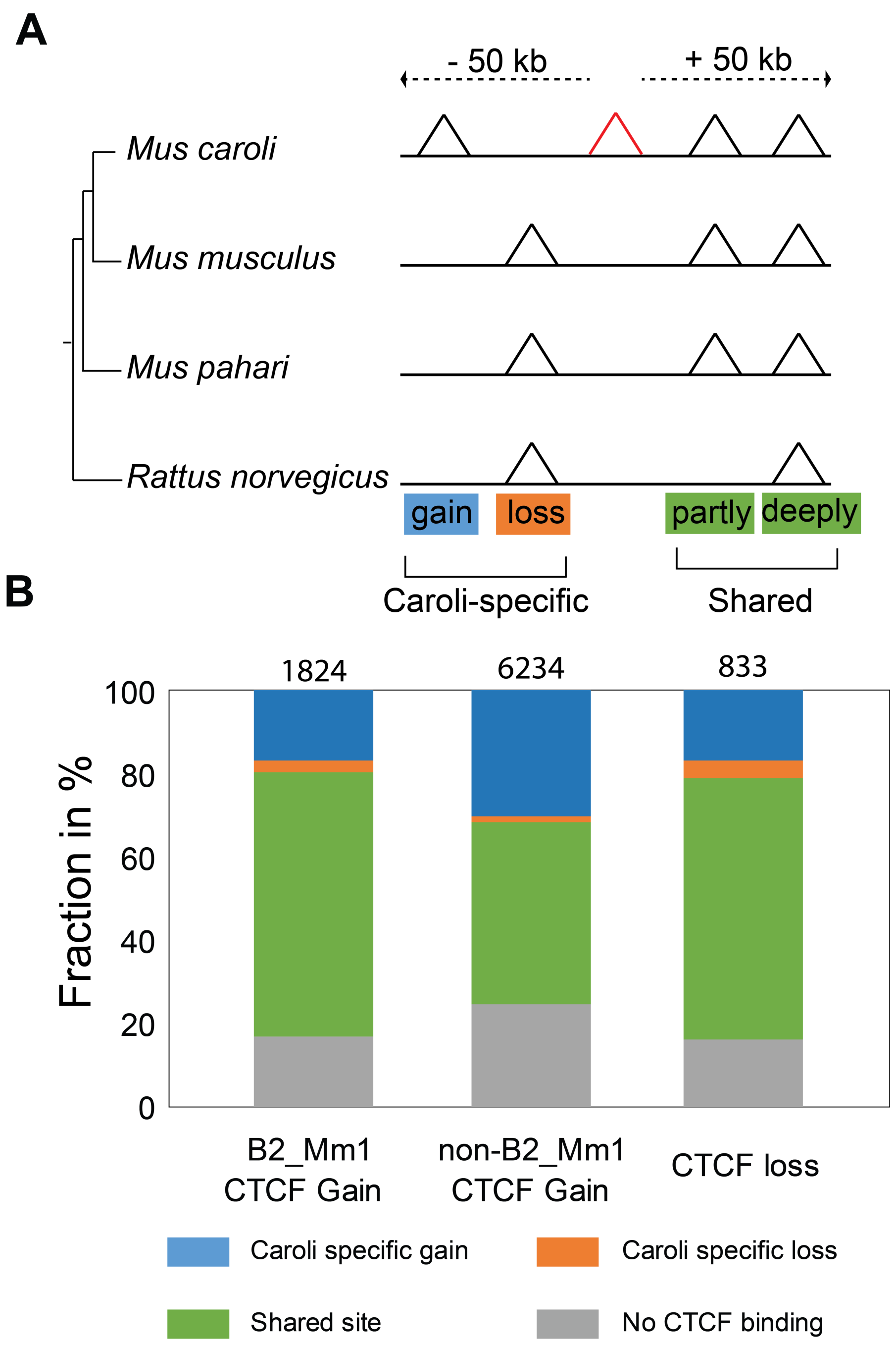

Figure S14. B2_Mm1 expanded CTCF binding occurs in CTCF rich regions.
A *Mus caroli* specific CTCF binding gains or losses (red central peak) are classified by whether they occurred close to (i) CTCF binding gains specific to *Mus caroli* (ii) CTCF binding losses specific to *Mus caroli*, or (iii) CTCF binding shared between *Mus caroli* and the other Muridae.
B Fraction of *Mus caroli* CTCF binding gains associated with the monophyletic B2_Mm1 repeat (left column) that are close to (i) *Mus caroli* specific CTCF binding gains (blue), (ii) *Mus caroli* specific CTCF binding losses (orange) (iii) *Mus caroli* CTCF binding shared with other Muridae (green) or no CTCF binding (grey). Middle column displays fractions not associated with a B2_Mm1 repeat and right column fractions associated with *Mus caroli* specific CTCF binding losses
