# Supplementary material for: Repeat associated mechanisms of genome evolution and function revealed by the Mus caroli and Mus pahari genomes
Source: Genome Res. 2018 Apr;28(4):448–59. doi: 10.1101/gr.234096.117 (PMC5880236; doi:10.1101/gr.234096.117)
Supplement: Supplemental Material [file supp_gr.234096.117_Supplemental_Methods.pdf]

Supplemental Material

Repeat associated mechanisms of genome evolution and function revealed by *the Mus caroli* and *Mus pahari* genomes

David Thybert[1,2], Maša Roller[1], Fábio C.P. Navarro[3], Ian Fiddes[4], Ian Streeter[1], Christine Feig[5], David Martin-Galvez[1], Mikhail Kolmogorov[6], Václav Janoušek[7], Wasiu Akanni[1], Bronwen Aken[1], Sarah Aldridge[5,8], Varshith Chakrapani[1], William Chow[8], Laura Clarke[1], Carla Cummins[1], Anthony Doran[8], Matthew Dunn[8], Leo Goodstadt[9], Kerstin Howe[3], Matthew Howell[1], Ambre-Aurore Josselin[1], Robert C. Karn[10], Christina M. Laukaitis[10], Lilue Jingtao[8], Fergal Martin[1], Matthieu Muffato[1], Stefanie Nachtweide[11], Michael A. Quail[8], Cristina Sisu[3], Mario Stanke[11], Klara Stefflova[5], Cock Van Oosterhout[12], Frederic Veyrunes[13], Ben Ward[2], Fengtang Yang[8], Golbahar Yazdanifar[10], Amonida Zadissa[1], David J. Adams[8], Alvis Brazma[1], Mark Gerstein[3], Benedict Paten[4], Son Pham[14], Thomas M. Keane[1,8], Duncan T Odom[5,8]*, Paul Flicek[1,8]*

[1] European Molecular Biology Laboratory, European Bioinformatics Institute, Wellcome Genome Campus, Hinxton, Cambridge, CB10 1SD, United Kingdom
[2] Earlham Institute, Norwich research Park, Norwich, NR4 7UH, United Kingdom
[3] Yale University Medical School, Computational Biology and Bioinformatics Program, New Haven, Connecticut 06520, USA
[4] Department of Biomolecular Engineering, University of California, Santa Cruz, California 95064, USA
[5] University of Cambridge, Cancer Research UK Cambridge Institute, Robinson Way, Cambridge CB2 0RE, UK
[6] Department of Computer Science and Engineering, University of California, San Diego, La Jolla, CA 92092
[7] Department of Zoology, Faculty of Science, Charles University in Prague, Prague, Czech Republic Institute of Vertebrate Biology, ASCR, Brno, Czech Republic
[8] Wellcome Sanger Institute, Wellcome Genome Campus, Hinxton, Cambridge, CB10 1SA, United Kingdom
[9] Wellcome Trust Centre for Human Genetics, Oxford, UK.
[10] Department of Medicine, College of Medicine, University of Arizona.
[11] Institute of Mathematics and Computer Science, University of Greifswald, Greifswald, 17487, Germany
[12] School of Environmental Sciences, University of East Anglia, Norwich Research Park, Norwich, United Kingdom
[13] Institut des Sciences de l'Evolution de Montpellier, Université Montpellier / CNRS, 34095 Montpellier, France
[14] Bioturing Inc, San Diego, California

Corresponding authors: DTO (duncan.odom@cruk.cam.ac.uk); PF (flicek@ebi.ac.uk)

Contents:

Supplemental Methods 1 – Sequencing, assembly, and annotation of *Mus caroli* and *Mus pahari* genomes (p4)

SM1.1 – Whole genome DNA library preparation, sequencing and scaffold assembly (p4)
SM1.2 – Optical mapping super scaffolds assembly (p4)
SM1.3 – *Mus pahari* inter-chromosomal break point identification (p5)
SM1.4 – *Mus pahari* and *Mus caroli* pseudo chromosomes assembly (p5)
SM1.5 – Genome assemblies used in this study (p6)
SM1.6 – RNA-seq data generation (p6)
SM1.7 – Gene annotation (p6)
SM1.8 – Coding gene orthologues identification (p7)
SM1.9 – Transposable element annotation (p7)
SM1.10 – Retrocopy annotation (p8)
SM1.11 – CTCF occupancy site identification (p8)
SM1.12 – Whole genome pairwise alignments (p9)
SM1.13 – Whole multiple genome alignment (p9)
SM1.14 – Quality check of genome assemblies (p10)
SM1.15 – Gene completion analysis (p10)
SM1.16 – Time divergence estimation (p10)
SM1.17 – Introgression analysis (p11)

Supplemental Methods 2 – A punctuated chromosomal rearrangement shaped the *Mus musculus* and *Mus caroli* ancestral karyotypes (p12)

SM2.1 – Pairwise genome alignment visualization (p12)
SM2.2 – Estimation of the rate of synteny break (p12)
SM2.3 – Repeat enrichment analysis in the chromosome breakpoints (p13)

Supplemental Methods 3 – Divergence and turnover of genomic sequences and segments are accelerated in Muridae, particularly for LINE retrotransposons (p14)

SM3.1 – Nucleotide variation rate estimation and comparison (p14)
SM3.2 – Segmental turnover rate estimation and comparison (p14)

Supplemental Methods 4 – Accelerated LINE retrotransposon activity has shaped coding gene evolution in rodents (p16)

SM4.1 – Estimation of the age of transposable elements (p16)
SM4.2 – Comparison of species-specific and ancestral repeat content (p16)
SM4.3 – Estimation of the age of retrocopies (p16)
SM4.4 – Identification of chimeric host genes fused with retrocopies (p17)
SM4.5 – Annotation of the *Abp* (*Scgb*) gene cluster (p17)
SM4.6 – Repeat enrichment analysis in the *Abp* gene cluster (p18)

Supplemental Methods 5 – A single nucleotide mutation transform a SINE B2 element in a CTCF carrier (p19)

SM5.1 – Classification of SINE B2 subfamilies (p19)

SM5.2 – CTCF representation in different repeat classes/families/subfamilies (p19)
SM5.3 – Age estimation of SINE B2 elements (p20)
SM5.4 – Comparison of SINE B2 subfamily representation between ancestral and species specific repeat set (p20)
SM5.5 – SINE B2_Mm1 neighbor-joining classification (p20)
SM5.6 – CTCF motif identification (p20)
SM5.7 – Ancestral motif inference (p20)
SM5.8 – CTCF trinucleotide analysis (p21)
SM5.9 – CTCF turnover analysis (p21)

References (p22)

Supplemental Methods 1 – *Mus caroli* and *Mus pahari* genome sequencing and assembly

SM1.1 – Whole genome DNA library preparation, sequencing and scaffold assembly

Genomic DNA was isolated from flash frozen tail samples from female *Mus caroli/EiJ* and *Mus pahari/EiJ* mice using either Invitrogen's Easy-DNA kit (K1800-01) or Gentra Puregene mouse tail kit (158267). The paired-end overlapping libraries were prepared following the ALLPATHS-LG recommended recipe (Gnerre et al. 2011). We sequenced each library on a HiSeq2000 with a read length of 100 bp. We followed established protocol to prepare 3 kb mate-pair libraries (Park 2013) and sequenced each end to read length 120 bp on a HiSeq2000.

For *Mus caroli* and *Mus pahari* we obtained in total 1.69 x 10^9 and 2.53 x 10^9 reads for the pair-end libraries and 1.95 x 10^9 and 2.46 x 10^9 reads for the mate-pair libraries, respectively. This yielded a theoretical coverage of ~135x for *Mus caroli* and ~185x for *Mus pahari*.

We assembled the Illumina reads of *Mus caroli* and *Mus pahari* into contigs and scaffolds using the ALLPATHS-LG assembler (Gnerre et al. 2011). The quality criteria of ALLPATHS-LG were satisfied by 67.2% of *Mus caroli* and 54.1% of *Mus pahari* paired-end reads; and 13.0% of *Mus caroli* and 10.3% of *Mus pahari* mate-pair reads. The assembly process yielded 31 317 scaffolds with a N50 of 195 kb for *Mus caroli*, and 19 269 scaffolds with a N50 of 331 kb for *Mus Pahari*.

SM1.2 – Optical mapping super scaffolds assembly

We assembled the scaffolds into super-scaffolds using an OpGen whole genome optical map from high molecular weight DNA. For both species, we extracted high molecular weight DNA from the spleens of female mice. After dissociation, spleen cells were embedded in agarose plugs (100ul) to minimize physical shearing of the extracted DNA and treated overnight with Proteinase K. The plugs were subsequently washed in TE (pH 8), melted, mixed with 100ul of an Agarose solution (4ul Agarose (1000U/ml)/96ul TE) and heated to 42°C for 12 hours. This process resulted in a high molecular weight DNA sample for each mouse species.

DNA was flowed down a microfluidic device into channels where it was linearized and fixed in position on the charged mapping surface. The MapCard chambers were then loaded with JOJO™ stain, a restriction enzyme (KpnI which cuts the mouse DNA every 6-12 kb), buffer and antifade. The card was then cycled on the Argus® MCP (MapCard Processing Unit) for approximately 25 minutes, where reagents are flowed over the DNA sample and digestion occurs at 37°C.

Following data collection, the mapset (total dataset) was filtered for minimum molecule size (>250 kb), minimum fragments per molecule (>12) and minimum molecule quality (>0.4). This resulted in 1 600 266 molecules for *Mus caroli* and 1 759 620 molecules *for Mus pahari*. We then used OpGen's Genome-Builder pipeline to assemble the genomic maps. Genome-Builder uses local single molecule assemblies from optical mapping to join sequence contigs together, creating large sequence scaffolds. The resultant local optical map

assemblies can span several megabases and provide previously absent long-range information. Taking this approach, we input all sequence contigs over 100 kb through iterative single molecule extension and mapped data into the sequence assembly gaps. We were able to generate 3386 alignments in the *Mus caroli* genome and 3161 alignments in the *Mus pahari* genome.

For *Mus caroli* and *Mus pahari,* we produced 3079 and 2944 super-scaffolds, respectively, including at least two contigs larger than 100 kb with a resulting N50s of 4.03 Mb and 3.67 Mb.

SM1.3 – *Mus pahari* inter-chromosomal break point identification

To identify potential inter-chromosomal rearrangements between the laboratory mouse *Mus musculus* C57BL/6J and *Mus caroli* or *Mus pahari*, we conducted reciprocal cross-species chromosome painting experiments to establish the genome-wide chromosome correspondence. We used short-term spleen cultures from C57BL/6J and *Mus caroli*, and an embryonic fibroblast cell line from *Mus pahari* to flow sort the chromosomes. Then we generated chromosome-specific DNA probes by degenerate oligo-primed PCR (for detailed protocols see (Yang and Graphodatsky 2017). To colour the chromosomes, mouse 21-colour painting probes were first hybridized onto the metaphase chromosomes of *Mus pahari or Mus caroli,* the painting probes derived from *Mus pahari* or *Mus caroli* were subsequently painted reversely onto the metaphase chromosomes of the C57BL/6J mouse.

To further define the evolutionary syntenic breakpoints on chromosomes of the C57BL/6J strain, the chromosome-specific DNA libraries of *Mus pahari* were pooled together according to breakpoints defined by chromosome painting, following the strategy previously used in the human and gibbon cross-species array-painting (Carbone et al. 2006) . Mouse CGH (244k) microarray slides were purchased from Agilent and array-painting followed the protocols supplied by the manufacturer. We analysed the array CGH using the CGHWeb tool (Lai et al. 2008).

SM1.4 – *Mus pahari* and *Mus caroli* pseudo chromosomes assembly

We assembled the super-scaffolds and scaffolds into pseudo-chromosomes using the Ragout method (Kolmogorov et al. 2016). This method uses multiple reference genomes of closely related species to guide the assembly of pseudo-chromosomes. Ragout shows significant improvement of the assembly compared to methods using one unique reference genome (Kolmogorov et al. 2016).

We generated a whole genome multiple alignment containing *Mus caroli*, *Mus pahari, Mus musculus* (C57BL/6NJ GRCm38/mm10 assembly) and with *Rattus norvegicus* V5.0 as an outgroup using the Progressive Cactus alignment tool (Paten et al. 2011). Then we applied the Ragout algorithm on the multiple alignments. For *Mus pahari* we added an inter-chromosomal rearrangement map to guide the assembly with *Mus musculus* due to inter-chromosomal rearrangements between these two species. The resulting genomes have 20 and 24 pseudo-chromosomes and a total size of 2.55 Gb and 2.47 Gb, respectively, for *Mus caroli* and *Mus pahari*.

SM1.5 – Genome assemblies used in this study

We used the genome assemblies described in the table for all subsequent analysis unless otherwise specified.

Clade	Species	Version
Muridae	*Mus musculus*	GRCm38
	Mus caroli	CAROLI_EIJ_v1
	Mus pahari	PAHARI_EIJ_v1
	Rattus norvegicus	Rnor_6.0
Hominidae	*Homo sapiens*	GRCh38
	Pan troglodytes	CHIMP2.1.4
	Gorilla gorilla	gorGor3.1
	Pongo abelii	PPYG2
	Macaca mulatta	MMUL_1
	Callithrix jacchus	C_jacchus3.2.1
Rodent	*Ictidomys tridecemlineatus*	SpeTri2.0

SM1.6 – RNA-seq data generation

RNA was extracted from multiple tissues (brain, liver, heart, kidney) from *Mus caroli* and *Mus pahari* using Qiagen's RNeasy kit following manufacturer's instructions. The RNA integrity was measured with the Agilent Bioanalyzer RNA nano chip and total RNA-seq libraries were generated with Illumina's TruSeq ribo-zero strand specific kit. All libraries were sequenced paired-end 100 bp on either Illumina's HiSeq2000 or HiSeq2500.

SM1.7 – Gene annotation

To annotate the genomes of *Mus caroli* and *Mus pahari* we used RNA-seq data from brain, heart, liver and kidney tissue (defined in **SM1.6**) and combined three annotation pipelines: TransMap (Stanke et al. 2008), AUGUSTUS (Stanke et al. 2006), and a new mode of AUGUSTUS called Comparative AUGUSTUS (AUGUSTUS-CGP) (Konig et al. 2016).

We started the annotation process by projecting the *Mus musculus* annotations produced by the GENCODE consortium (M8 release) (Harrow et al. 2012) to *Mus caroli* and *Mus pahari* assemblies using TransMap and the Progressive Cactus multiple alignment defined in **SM1.4**. Protein-coding transcript projections were used as strong hints to the gene finding tool AUGUSTUS along with subspecies-specific RNA-seq data for both mouse species. Next, we used AUGUSTUS-CGP that makes use of the whole genome alignment along with RNA-seq information and annotation information to simultaneously predict

protein-coding transcripts in all aligned genomes. AUGUSTUS-CGP integrates the evolutionary dependencies between the genomes and the individual gene models on each genome. The output is a consistent set of transcripts across the genomes of *Mus caroli* and *Mus pahari*.

The transcript projections, AUGUSTUS transcripts, and AUGUSTUS-CGP transcripts were combined through a consensus finding process into an annotation set for each species. AUGUSTUS-CGP transcripts were assigned putative parental genes based on coordinate overlaps. The consensus finding algorithm then used a combination of a series of binary classifiers that evaluate transcript fidelity and transcript-transcript alignment metrics to determine the highest quality transcript to represent the orthologuous transcript. Each transcript was assigned a category of excellent, passing or failing. If all transcripts for a gene are categorized as failing, one longest transcript was picked to represent the locus. A series of heuristics were used to resolve paralogous alignments and genes split across multiple chromosomes. If an AUGUSTUS-CGP transcript was not assigned to an existing annotation, it was designated as a putative novel transcript. AUGUSTUS-CGP transcripts with novel splice junctions supported by RNA-seq that overlapped existing annotations were assigned as novel isoforms of that existing gene.

In total, 103 889 transcripts representing 32 370 genes were annotated in *Mus caroli* and 100 787 transcripts representing 31 212 genes in *Mus pahari*. Identified protein-coding genes numbered 20,323 in *Mus caroli* and 20,029 in *Mus pahari*. AUGUSTUS-CGP identified 1,470 transcripts with novel splice sites in *Mus caroli* and 1,550 transcripts with novel splice sites in *Mus pahari*. 26 genes were not assigned as orthologues to *Mus musculus* in *Mus caroli*, and 28 genes in *Mus pahari*.

For the mouse reference and Hominidae genomes we used the Ensembl v80 (Cunningham et al. 2015).

SM1.8 – Coding gene orthologues identification

To identify coding-gene orthologues between a pair of species, we first used the APPRIS database (Rodriguez et al. 2013) to select the primary coding transcript in the *Mus* species defined in **SM1.5**. For each gene annotated as an orthologue we identified the highest ranked APPRIS transcript for which we had a confident coding interval with no frame-shifting indels and proper start/stop codons. In addition, we filtered out genes which had any paralogous alignments.

For the Hominidae species and the *Mus musculus* / *Rattus norvegicus* pair we used one-to-one orthologues defined in Ensembl v80.

SM1.9 – Transposable element annotation

To identify the transposable elements in the Muridae and Hominidae genomes defined in **SM1.5** we first used RepeatMasker 3.2.8 (A.F.A. Smit, R. Hubley & P. Green RepeatMasker at http://repeatmasker.org) using the rodent repeat library for the Muridae and the primate repeat library for Hominidae. Then we applied post-processing steps on the RepeatMasker output to filter out non-transposable repeats and spurious hits: we removed all

the simple repeats and microsatellite elements to keep only the transposable elements. In addition, we merged fragmented hits from the same repeat element identified by RepeatMasker. After these post-processing steps, we found between 3 306 375 and 3 727 882 transposable elements for the Muridae and between 2 850 536 and 4 330 735 for the primate genomes. We used this set of repeats for the following analyses unless otherwise specified.

SM1.10 – Retrocopy annotation

Retrocopies are intronless copies of protein-coding genes lack introns because they are created through retrotransposition of the genes' mRNAs. Retrocopies in the Muridae and primate genomes were detected as previously described (Navarro and Galante 2013). We considered the manually-annotated processed pseudogenes from GENCODE M13 (mouse) and v24 (human) respectively (Pei et al. 2012) and processed pseudogenes from pseudopipe (Zhang et al. 2006; Sisu et al. 2014). Mature transcript sequences were derived from Ensembl v86 and aligned to the corresponding reference genome using BLAT (mask=lower; -tileSize=12; -minIdentity=75; -minScore=100). We selected the BLAT hits that had greater than 75% identity to the parental mature transcript, and either coverage higher than 50% or at least 120 nucleotides aligned to the reference genome. We filtered out (i) hits that had less than two merged exons (i.e exons lacking the intron in between), (ii) hits that had at least a 15 kb gap between exons and (iii) hits that were close to their original parental gene. In total, we found between 6912 and 9546 retrocopies in Muridae and 7499 and 11 153 retrocopies in the six primate genomes defined in **SM1.5**.

SM1.11 – CTCF occupancy site identification

To identify CTCF occupancy sites in Muridae we performed chromatin immunoprecipitation experiments followed by deep sequencing (ChIP-seq) in *Mus caroli/EiJ* (Jackson Laboratory stock number 000926) and *Mus pahari/EiJ* (Jackson Laboratory stock number 002655) individuals and C57BL6/J mice (purchased from Charles River). We maintained the colonies under specific-pathogen-free conditions with food and water ad libitum in accordance with the Animals (Scientific Procedures) Act 1986 and recommendations of the animal ethics committee of Cancer Research UK and the University of Cambridge. CTCF ChIP-seq data for *Rattus norvegicus* was previously published (Schmidt et al. 2012).

Liver tissue from three male individuals from the four species was crosslinked, dounced and lysed as previously described (Schmidt et al. 2009). The chromatin was sonicated using a Misonix sonicator 3000 homogenizer with 12 cycles of 30s ON and 60s OFF at a power-output 30 Watt. CTCF was immunoprecipitated using the Merck-Millipore antibody (cat# 07-729) and NGS libraries prepared as previously described (Schmidt et al. 2009) ligating Illumina's low-plex TruSeq adapters. Libraries were quantified using the KapaBiosystems library quantification kit, pooled and sequenced at 100 bp paired-end on the HiSeq2000 platform.

Sequencing reads were aligned to the appropriate reference genome (genome description in **SM1.5**) using Bowtie 2 version 2.2.6 and the parameters "--very-sensitive -N 1 --seed 1" (Langmead and Salzberg 2012). Regions of read enrichment (peaks) were called

using MACS version 1.4.2 (Zhang et al. 2008) and appropriate matched input libraries as control samples. For all libraries, a tag size (-s option) of 200 and a p-value (-p option) threshold of 5e-3 were used. An effective genome size (-g option) of 2.4e9 was used for the rat and for all *Mus* species a size of 1.87e9. Peaks were called per biological replicate, but only those peaks that appeared in two or more replicates were kept for further analyses. To identify CTCF occupancy sites in primates we used previously published datasets (Schwalie et al. 2013) and the same methods as described above, but using an effective genome size of 2.7e9.

In total, we found between 29 924 and 48 153 CTCF binding sites in Muridae and between 41 656 and 66 281 CTCF binding sites in the Hominidae.

SM1.12 – Whole genome pairwise alignments

We computed pairwise alignments using LastZ following Ensembl's methodology on the Muridae genomes defined in **SM1.5** (Herrero et al. 2016). Ensembl LastZ alignments are directional, and the table below lists the three reference genomes used and the non-reference genomes they were aligned to (an empty cell means that the alignment was not computed). Two sets of parameters were used, depending on the expected similarity of the genomes. In the table, "P" indicates comparisons performed with parameters tuned for closely-related genomes and "O" comparisons performed between more divergent genomes. The actual parameters are listed in the table 1 of (Herrero et al. 2016), respectively as "Primates" and "Other" parameters.

Table ST1.1 : Pairwise alignment performed and list of parameters used.

	CAST_EiJ_v1	SPRET_EiJ_v1	CAROLI_EIJ_v1	PAHARI_EIJ_v1	Rnor_6.0
GRCm38	P	P	P	O	O
Rnor_6.0	O	O	O	O	
CAROLI_EIJ_v1				P	

For the Hominidae genomes we used the pairwise alignments from Ensembl v80.

SM1.13 – Whole multiple genome alignment

We used the Enredo-Pecan-Ortheus pipeline (Hubbard et al. 2009) to align the Muridae genomes defined in **SM1.5** with all mammalian genome assembled at the chromosome level and present in Ensembl v80. We first aligned with exonerate (Slater and Birney 2005; Herrero et al. 2016) a set of 7 400 783 million anchors (DNA sequences of average length: 102 bp) conserved across 9 species (representing 883 096 orthology groups). Enredo (Paten et al. 2008a; Herrero et al. 2016) then defined synteny blocks from a synteny graph built using the positions of these alignments. Each block was aligned with Pecan (Paten et al. 2008a) and then Ortheus (Paten et al. 2008b). This yielded 294 574 alignment blocks total (average length: 96 253 bp), which covered on average 82.1% of each genome.

SM1.14 – Quality check of genome assemblies

In order to assess the quality of the genome assemblies we used different strategies to estimate the fractions of the genomes that were potentially misassembled. First, we estimated the nucleotide error rate by calling single nucleotide variation between the assembly and the 3 kb mate-pair libraries that have been used only to assemble contigs into scaffolds (i.e they had not been used to generate the sequence of contigs). We mapped the libraries with BWA-MEM using default parameters (Li and Durbin 2010). We removed PCR duplicates using SAMtools and ignored low quality mapped reads (MQ <30). Then we called single nucleotide variation using GATK with the default parameters (McKenna et al. 2010). The number of single nucleotide variations found gave the estimate of the error rate.

Next we sought to find assembly error by finding structural variants between the current assemblies and optical maps. We created a consensus between the assembly and raw optical map reads using the Genome-Builder suite from OpGen. We compared the inconsistency between the map and the assembly. We called an insertion when a genomic fragment was present in the assembly but absent in in the optical map and we called a deletion when a genomic fragment was absent in the assembly, but present in the optical map. We calculated the cumulative size of insertions and deletions to estimate the size of misassembled regions.

SM1.15 – Gene completion analysis

We used the Benchmarking Universal Single-Copy Orthologs (BUSCO) (Simao et al. 2015) gene set to assess the gene completeness of the assemblies. We used the vertebrate data set (3023 genes) in BUSCO v1 and aligned the protein sequences against *Mus pahari* and *Mus caroli* genomes. Ensembl vertebrate genomes were used as the reference set. All analyses were performed on repeat masked assemblies, using RepeatMasker as defined in **SM1.9**. The alignments were computed using exonerate (Slater and Birney 2005), with the below parameters: --model protein2genome --forwardcoordinates FALSE --softmasktarget TRUE --exhaustive FALSE --bestn 1. The average coverage for each species was then calculated based on all the BUSCO genes that had alignments against that genome.

SM1.16 – Time divergence estimation

To estimate the divergence time that separates *Mus musculus* with *Mus caroli* and *Mus pahari we* first created a dataset of near neutral evolving loci. We selected the 584 985 four-fold degenerated sites from conserved amino acids across all mammals included in the multiple alignment described in **SM1.13**. We considered these sites to evolve near neutrally in the murine clade. Then we used this set of four-fold degenerate sites to create three different subsets with an equal number of four-fold degenerated sites:

1- randomly selected sites

2- sites from genes with tissue specific expression (genes expressed in only one tissues from the list: blood, liver, pancreas, hypothalamus, testis)

3- sites from housekeeping genes (expressed in at least 12 tissues).

We used the gene expression data from (Su et al. 2004) with the accession E-MTAB-25 in European Nucleotide Archive to establish tissues specific and housekeeping genes as described above.

Next we inferred the divergence time separately for each dataset (random, tissues-specific, housekeeping) using the software BEAST 2 (Bouckaert et al. 2014). Independent inference of divergence time from the three datasets was performed to control for each category's effect on the estimation procedure.

We used different models for the speciation process (calibrated Yule model, Birth–Death Model), the nucleotide mutation process (GTR, HKY85) and for variation of mutation rate (strict clock, uncorrelated relaxed clock). For the strict clock model we used a gamma distribution as prior using the following parameters (α=0.001, β=1000, offset=0, initial=1, range= [$-\infty$,∞]). For the uncorrelated relaxed clock model, we used the default parameters for calculating the mean and used an exponential distribution (mean=0.333, initial=0.5, range= [0, 5]) for calculating the standard deviation. We used the default parameters for all other models. In total 24 different analyses combining the different models and sets of neutral evolving sites were performed.

We used fossil record information of the mouse-rat divergence to calibrate the molecular clock in all our analyses. We used a log normal distribution (mean = 0.8, sd = 0.55 and offset = 10.4) to define the divergence probability of mouse rat. This distribution was based on the facts that the first murine (*Potwarmus* sp) was dated to 18.5 MYA (Lopez Antonanzas 2009), the consensus for the time divergence between *Mus* and *Rattus* is around 12 MYA (Jacobs and Flynn 2005), and the first fossil of the Rattus ancestor (*Karnimata sp.*) was dated to 10.4 MYA (Jacobs and Flynn 2005).

The resulting trees for each analysis were summarized with the TreeAnnotator tool from the BEAST suit. The final tree used to draw Fig. 1 was defined with maximum clade credibility as the target tree type and the median of node heights and was edited with FigTree (version 1.4.2, http://tree.bio.ed.ac.uk/software/figtree).

SM1.17 – **Introgression analysis**

To detect introgression signal between *Mus musculus* and *Mus caroli* or *Mus pahari*, we used HybridCheck (Ward and van Oosterhout 2016) on the multiple genome alignment defined in **SM1.13** including *Mus musculus* strain C57B6, *Mus castaneus, Mus caroli* and *Mus pahari*. We used *Mus castaneus* as a positive control for a genome that can introgress with *Mus musculus* (Keane et al. 2011). We found less than 1% of the genome as introgressed between *Mus musculus* and *Mus caroli* or *Mus pahari*. By comparison we found around 12.5% of the *Mus castaneus* genome as introgressed with *Mus musculus*, meaning that *Mus caroli* and *Mus pahari* have virtually no introgressed regions with Mus musculus.

Supplemental Methods 2 - A punctuated chromosomal rearrangement shaped the *Mus musculus* and *Mus caroli* ancestral karyotypes

SM2.1 – Pairwise genome alignment visualization

To visualize inter-chromosomal rearrangements between a pair of species, we used the pairwise genome aligner MUMmer 3 (Kurtz et al. 2004). MUMmer enables generation of multi-chromosome alignments with the tool mummerplot. We computed pairwise alignments of all chromosomes from one species against all chromosomes in the other species. We set the parameters maxgap at 2000 and mincluster at 1000. We then used the delta-filter tool from the MUMmer suit to filter out small alignments by setting the parameters -l at 1000000 and -g. Finally, we used the mummerplot tool to generate the multi-chromosome plot. We modified the order of the chromosomes manually in the delta file in order to maximize the linearity against the diagonal of the multi-chromosome plot.

SM2.2 – Estimation of the rate of synteny break

We identified synteny breaks involving large genomic regions between *Mus musculus* and *Mus pahari* by using the chromosome painting experiment (**SM1.3**). All synteny breaks found with this approach involved chromosome regions larger than 3 Mb. Consequently, we set the resolution of the chromosome painting approach to 3 Mb. To identify chromosomes synteny breaks between *Mus musculus* and the rat and all Hominidae genomes, we used the synteny map in Ensembl v80. We selected chromosomal synteny breaks between the different species using regions larger than 3 Mb to be consistent with the chromosome painting approach. We did not take into account chromosome inversions or intra-chromosome translocations in order to be consistent with the chromosome painting approach that cannot identify such events.

We used a lenient and a strict approach to count the number of synteny breaks between a pair of species. The lenient approach counted all synteny breaks in the inter-chromosomal rearrangement. The strict approach counted the number of different chromosomes involved – 1. In the following example:

Rat_chr9 = mm_chr17:38.4-57.5 + mm_ chr1:18.1-97.6 + mm_ chr17:58.9-71.6

the lenient approach will count 2 synteny breaks and the strict will count only 1. The strict approach considered that an intra-chromosome translocation may be involved when a chromosomal rearrangement involves the same chromosome twice. The total number of synteny breaks for a pair of species is defined as the number of synteny breaks in one comparison (for example mouse vs rat) and the number of synteny breaks in the reciprocal comparison (for example rat vs mouse).

To estimate the rate of synteny breaks within the branch of each clade (i.e Muridae and Hominidae), we counted the number of synteny breaks (lenient and strict approaches) between all pairwise comparison of species within a clade. For each clade, we constructed a distance matrix for which the distance between a pair of species is defined as the number of synteny breaks. We used the distance matrix to compute a neighbor-joining tree for which the distance of each branch is an estimation of the number of synteny breaks that happen in the

branch (**Fig. 1C**). To estimate the rate of synteny breaks within a branch, we divided the number of synteny breaks within a branch by the length of the branch in MY. The error bar of the rate was defined by using a 5% error rate bar of the estimation of divergence time between each species. The results were comparable whether the analysis was done with the strict or lenient approach, and only the strict comparison is plotted.

SM2.3 – **Repeat enrichment analysis in the chromosome breakpoints**

To propose a potential mechanism driving the punctuated event of chromosome rearrangements that occurred between 3 and 6 MYA in the Muridae, we searched for enrichment of repeat elements in the vicinity of the inter-chromosomal breakpoints between *Mus musculus* and *Mus pahari*. We defined two different repeat categories from the dataset described in **SM1.9**: (i) a dataset including all retrotransposons and transposons and (ii) a dataset including retrotransposons and transposons with an age between 3 and 6 MY.

To identify the 3-6 MY old transposons and retrotransposons we took advantage of the fact that the sequence of transposable elements evolves nearly neutrally and that the sequence divergence of the repeat with its ancestral sequence is linearly related to the age of the repeat. We used the consensus sequence of the repeat subfamilies as a proxy for the ancestral repeats and evaluated the sequence identity between the repeat element and its consensus sequence. We estimated the evolutionary rate of nucleotide divergence in ancestral repeats shared by the four Muridae species and selected all repeat elements that had a sequence divergence between 3.5% and 7% with the consensus sequence, corresponding to repeat elements with an age between 3 and 6MY.

Next we took the chromosome breakpoints between *Mus musculus* and *Mus pahari* defined in **SM1.3** and looked for enrichment of repeat elements using a 200 kb sliding window in a region of +/-40 Mb around the breakpoints. We calculated a z-score of the repeat density based on the mean and the standard deviation of the 200 kb bin of the whole region excluding the region +/-2 Mb around the breakpoint. We plotted the smoothed repeat density using a sliding window approach by averaging the value of the five upstream bins and first five downstream bins (**Supplemental Fig. S5)**

To evaluate the statistical significance of the repeat enrichment we calculated the observed number of repeat elements in the +/-2 Mb genomic region around the 14 chromosomal breakpoints. Then we evaluated the expected number of repeats in the genome by calculating the number of repeat elements in 14 random regions of the genome. We repeated this process 1,000,000 times in order to define the distribution of the expected values. We derived an empirical p-value by comparing the observed value with the distribution of the expected values for of each class of transposable elements from the two repeat datasets.

Supplemental Methods 3 – Divergence and turnover of genomic sequences and segments are accelerated in Muridae, particularly for LINE retrotransposons

SM3.1 – Nucleotide variation rate estimation and comparison

To estimate the rate of nucleotide variation of different genomic categories in the Muridae and Hominidae we first classified the mouse and human reference genome into the following different fractions: (i) four-fold degenerate sites from conserved amino across mammals; (ii) ancestral LTR, SINE, LINE and DNA repeats defined as shared between the four Muridae or Hominidae; (iii) intergenic sequence defined as a genomic region between two annotated genes (protein-coding and non-coding genes); (iv) intronic sequence defined as the introns of the protein-coding genes; (v) exonic sequence defined as the exon of protein-coding genes; (vi) CTCF binding regions defined as the CTCF occupancy regions that have been identified with the ChIP-seq analysis pipeline described in **SM1.11;** (vii) CTCF binding motif defined as all CTCF binding motifs found in CTCF binding regions using FIMO program with default parameters from the MEME suite version 4.10.2 (Kurtz et al. 2004) and the CTCF position weight matrix (CTCF.p2) from the SwissRegulon database (Pachkov et al. 2013).

We then used the pairwise genome alignments described in **SM1.12** to calculate the sequence divergence between *Mus musculus* and all other Muridae and between human and all other Hominidae. For each clade, we plotted the values of the nucleotide divergence against the divergence time between *Mus musculus* and the other Muridae or human and the other Hominidae. We then applied a linear regression for each category and each clade and defined the rate of nucleotide divergence as the value of the slope of the line.

To compare the statistical significance of the difference of rates between each genomic category within a clade we applied an ANCOVA test. To compare the evolutionary rate of one genomic category between two clades we calculated the ratio $rate_{Muridae}$ / $rate_{Hominidae}$.

SM3.2 – Segmental turnover rate estimation and comparison

To estimate the rate of segmental turnover variation in different genomic categories of Muridae and Hominidae genomes, we first classified the genomes of each species in the two clades into the following categories: (i) protein-coding genes, (ii) DNA transposons, (iii) LTR retrotransposons, (iv) LINE retrotransposons and (v) SINE retrotransposons.

For each clade, we then used the pairwise genome alignments defined in **SM1.12** to estimate the fraction of shared genomic regions between a pair of species. We defined as a genomic region shared between two species all regions in one species that had an alignment in the other species with less than 50% of gapped sequence. For the protein-coding gene category, we considered as shared if the pair of species were sharing a functional orthologue (as defined in **SM1.8**). We calculated the fraction of unshared genomic regions between a pair of species A and B by calculating the mean of unshared genomic regions defined in a two-way comparison: A against B and B against A.

For each clade, we plotted the fraction of unshared genomic regions against the divergence time between *Mus musculus* and the other Muridae or human and the other Hominidae. We then applied a linear regression for each category and each clade and defined the rate of segmental turnover as the value of the slope of the line. We applied an ANCOVA test to evaluate the statistical significance of the difference of rates between each genomic category within a clade

Supplemental Methods 4 – Accelerated LINE retrotransposon activity has shaped coding gene evolution in rodents

SM4.1 – Estimation of the age of transposable elements

We estimated the age of the transposable elements defined in the **SM1.9** by calculating sequence identity between the transposable elements and their subfamily consensus sequence, which is an approximation of the ancestral repeat. Since the sequence of transposable elements evolves nearly neutrally, we can define a linear relationship between the sequence identity and the estimated age of a repeat.

We used the evolutionary rate of the four classes of transposable elements calculated in **SM3.1** to convert the sequence identity between the transposable element and it ancestral sequence into MY using the following linear relationship:

$$t = d / r$$

Where t is the divergence time between a repeats and its ancestral sequence , d is the sequence divergence between the repeat and its sequence ancestral sequence and r is the evolutionary rate of the sequence of the given class of transposable element calculated in **SM3.1.**

SM4.2 – Comparison of species-specific and ancestral repeat content

We compared the composition of transposable elements (TE) between the set of ancestral TE and species-specific TE in Muridae and Hominidae. We defined the set of ancestral Muridae/Hominidae TEs as the TEs from **SM1.9** that were shared between all four Muridae/Hominidae genomes in the multiple alignment defined in **SM1.13**. For each species, we defined a set of species-specific TEs as the TEs that were shared with no other species within each clade in the multiple alignment defined in **SM1.13**. The ancestral dataset was composed of 939,727 TEs for the Muridae and 606 473 TEs for the Hominidae. The species-specific datasets contain between 949 119 and 2 057 208 TEs for the Muridae and between 402 849 and 1 057 733 TEs for the Hominidae.

We compared the composition of TEs within classes/families/subfamilies between the datasets of ancestral and species-specific TEs and tested the statistical significance of the difference with Fisher's exact test.

SM4.3 – Estimation of the age of retrocopies

We estimated the age of the retrocopies defined in **SM1.10** by calculating the sequence identity between the retrocopies and their parental genes. The parental gene can be considered as a special ancestral sequence of retrocopy that has a sequence that evolved with an evolutionary rate r_{parent} from the time of the retrocopy creation. During the same period of time the retrocopies evolved nearly neutrally with an evolutionary rate $r_{retrocopy}$.

If we assume a linear relationship between the evolutionary sequence rate of the parental gene and divergence time at the clade level, then sequence divergence d can be defined as:

$$d=(r_{parent} + r_{retrocopy})/2 \times t. \qquad (1)$$

where t is the divergence time between the retrocopy and the parental gene. From (1) we can derive:

$$t=2d/(r_{parent} + r_{retrocopy}) \qquad (2)$$

We calculated the rate of sequence divergence of retrocopies based on the ancestral retrocopies i.e retrocopies that were shared between all four Muridae/Hominidae. For the rate of sequence divergence of the parental gene we used the rate for coding exons as defined in **SM3.1**

SM4.4 – Identification of chimeric host genes fused with retrocopies

To detect chimeric transcripts, we first used Bowtie 2 with default parameters to map to the respective genome the RNA-seq data **defined in SM1.6** and the RNA-seq data from C57BL6 brain, heart, liver, kidney generated by the ENCODE project (Yue et al. 2014). The data sets used were ENCSR554PHF, ENCSR164BAZ, ENCSR216KLZ, and ENCSR216KLZ in GEO.

Next, we selected alignments where one paired-end read overlapped loci annotated as retrocopies and the other paired-end read aligned to the host gene (i.e the gene that integrated the new retrocopy element). We did not consider alignments in which both reads overlapped the retrocopy and we excluded exonic retrocopies from this analysis. Next we assembled the putative transcripts de novo with Trinity (Haas et al. 2013) using default parameters by inputting the reads associated with the selected alignments and reads associated with 10 kb flanking regions. The exon-exon junction quantification and sashimi plots were based on STAR realignments of the mate-pair reads overlapping the host gene.

SM4.5 – Annotation of the *Abp* (*Scgb*) gene cluster

We annotated the *Abp* gene cluster in the 1504 assembly of *Mus caroli* and *Mus pahari* genomes. The 1504 build is a previous assembly version using the mouse reference genome to guide the scaffold and super-scaffold assembly into chromosomes. The CAROLI_EIJ_v1 and PAHARII_EIJ_v1 assembly versions have better overall assembly statistics than the 1504 build, but we found using manual inspection that more *Abp* genes were correctly assembled in the gene cluster of the 1504 build. We used a combination of BLAST (Altschul et al. 1990), hmmsearch (Finn et al. 2011) and Exonerate (Slater and Birney 2005) to find the *Abp* gene cluster based on the 64 *Abp* genes from the mouse reference genome (Emes et al. 2004; Laukaitis et al. 2008) and the three *Abp* genes from the rat reference genome (Emes et al. 2004). We obtained 17 *Abpa* genes and 16 *Abpbg* genes on *Mus caroli* chromosome 7, and 6 *Abpa genes* and 5 *Abpbg* genes on *Mus pahari* chromosome 1. We then searched again with the newly-identified sequences, and did not find any novel identifications. The sequences identified by the methods described above were searched

manually for start and stop codons and for donor and acceptor intron splice sites. We also verified the flanking genes *Scn1b* and *Uba2* (formerly *Uble1b*).

SM4.6 – Repeat enrichment analysis in the *Shbg* gene cluster

We analysed the density of retrotransposons in the *Abp* gene cluster of *Mus musculus* and rat with the repeat dataset described in **SM1.9**. For *Mus caroli* and *Mus pahari*, we used a repeat dataset based on the same approach described in **SM1.9** but applied on the on the 1504 builds (**See SM4.5**).

We counted the cumulative size of each class of retrotransposon in 50 kb bins in a +/- 5 Mb region from the centres of the *Abp* gene clusters. To estimate the statistical significance of the retrotransposons enrichment/depletion we calculated an empirical p-value using two approaches.

In the first approach, we randomly selected from each species' genome an equal number of 50 kb bins to the number of bins in the *Abp* gene cluster and 10 kb flanking regions. We iterated this procedure 1 000 000 times to define the distribution of random expected density in the flanking regions of the cluster. This distribution was then used to derive an empirical p-value.

In the second approach, we randomly selected from *Mus musculus* and the rat genome an equal number of 50 kb bins to the number of bins in the *Abp* gene cluster from randomly selected single genes, i.e. genes that have no paralogs in the genome. We iterated this procedure 1 000 000 times to define the distribution of random expected density in single genes. This distribution was then used to derive an empirical p-value.

Supplemental Methods 5 – A single nucleotide mutation transform a SINE B2 element in a CTCF carrier

SM5.1 – Classification of SINE B2 subfamilies

To classify the SINE B2 retrotransposons into subfamilies, we selected all SINE B2 retrotransposons from the Muridae dataset defined in **SM1.9**. These retrotransposons were multiply aligned using Clustal Omega version 2.1 with default parameters (Sievers et al. 2011). Those retrotransposons that had more than 95% pairwise identity were iteratively clustered together into groups. The resulting sequence clusters were then re-aligned using Clustal Omega and their consensus sequence computed with the cons program of the EMBOSS suite with default parameters (Rice et al. 2000). For further analyses the resulting five consensus sequences were used to represent rodent SINE B2 subfamilies

SM5.2 – CTCF representation in different repeat classes/families/subfamilies

We analysed the distribution of experimentally determined CTCF occupancy (**SM1.11)** in different classes, families and subfamilies of transposable elements. We first estimated the expected frequency of CTCF occupancy sites overlapping a repeat class/family/subfamily in each species. We randomly sampled in each genome the same number of regions with size matching the experimental CTCF occupancy sites. We calculated the frequency of regions overlapping a repeat element from the dataset defined in **SM1.9**. We reiterated this process 100,000 times and calculated the mean to define the expected frequency. Next, we calculated the observed frequency of CTCF occupancy sites overlapping a repeat class/family/subfamily using the same repeat dataset as defined in **SM1.9**. For each repeat class/family/subfamily, we evaluated the level of over/under-representation of CTCF occupancy in repeat classes/families/subfamilies by subtracting the expected frequency from the observed frequency. To evaluate the statistical significance of the difference between the observed and expected frequencies, we derived an empirical p-value by comparing the observed frequency with the distribution of expected frequencies using the ecdf function in R. The empirical p-value for overrepresentation of CTCF occupancy in the SINE B2 family, compared to other SINE families, is 0 for all rodent species investigated. The empirical p-value for overrepresentation of CTCF occupancy in the SINE B2 Mm1 subfamily, compared to other SINE B2 subfamilies, is: 0.000798 for *Mus musculus C57BL6*, 0 for *Mus caroli*, 1 for *Mus pahari*, and 0.410 for the rat.

We plotted the level of over/under-representation of CTCF occupancy sites overlapping repeat elements compared to the random expectation using the heatmap.2 function of the R gplots package. For the distribution of CTCF occupancy sites among repeat classes (**Fig. 4B**) only the transposable elements closest to the CTCF peak summit were used. For all other analyses, the transposable elements overlapping a CTCF occupancy site were used.

SM5.3 – Age estimation of SINE B2 elements

To estimate the age of SINE B2 elements we used the same approach as defined in **SM4.1.** We used the nucleotide evolution rate of all SINE elements to estimate the correspondence between the nucleotide sequence divergence and the divergence time.

SM5.4 – Comparison of SINE B2 subfamily representation between ancestral and species specific repeat set.

To compare between the sets of ancestral and species-specific SINE B2 elements, we used the same approach defined in **SM4.2**

SM5.5 – SINE B2_Mm1 neighbor-joining classification

To build a neighbor-joining tree of the SINE B2_Mm1 retrotransposons we first computed multiple alignments of SINE B2_Mm1 sequences selected from the *Mus* clade genomes defined in **SM1.9.** To reduce alignment error leading to erroneous clustering during the tree-building process, we filtered out all SINE B2_Mm1s with the following characteristics: (i) shorter than 150 bp; (ii) at least one unknown nucleotide (N); and (iii) more than 10% of substitution, insertion or deletion with the SINE B2_Mm1 consensus sequence. This filtering process removed 21% of the sequences leaving a total of 67,637 SINE B2_Mm1 retrotransposons from the three *Mus* species.

The sequences were aligned using MAFFT version 7.222 (Katoh and Standley 2013) with the options --nuc --reorder --retree 1 --op 20 --ep 4. The tree was calculated using FastTree version 2.1.9 (Katoh and Standley 2013) with local bootstrap and minimum-evolution criterion for speed (all options used were: -fastest -nosupport -noml -pseudo -nt). The resulting tree was plotted in iTol version 3.4.2 (Letunic and Bork 2016) with nodes coloured according to the species the SINE B2_Mm1 was originally extracted from and experimentally determined CTCF occupancy annotated as an outer circle.

SM5.6 – CTCF motif identification

To identify the CTCF binding motif in each binding region we used the FIMO program with default parameters from the MEME suite version 4.10.2 (Bailey et al. 2015) and the CTCF position weight matrix (CTCF.p2) from the SwissRegulon database (Pachkov et al. 2013).

SM5.7 – Ancestral motif inference

We used two approaches to infer the ancestral CTCF binding motifs in different branches of the SINE B2_Mm1 neighbor-joining tree defined in **SM5.5**. First the motifs found by FIMO in each transposable element of the predominantly CTCF binding branch and the adjacent predominantly non-binding branches were extracted. Next, we used FastML (Ashkenazy et al. 2012) with the neighbor-joining method and JC nuclear model of substitution to reconstruct the ancestral sequence of the resulting binding motifs. In the

second approach, we used PRANK (Ashkenazy et al. 2012) with the options -showanc -keep -njtree to reconstruct the ancestral motif. Both methods reconstructed the same ancestral sequence for the predominately CTCF binding and non-binding clusters.

SM5.8 – CTCF trinucleotide analysis

To assess whether the single nucleotide mutation T→C in the B2_mm1 ancestral CTCF binding motif at position 18 increased the binding intensity we counted the number of occurrences of all possible trinucleotides in the motif starting from position 18 in motifs bound and non-bound by CTCF in *Mus caroli*. We scanned the whole *Mus caroli* genome with the MEME suite version 4.10.2 FIMO program (Kurtz et al. 2004) and the CTCF position weight matrix (CTCF.p2) from the SwissRegulon database (Pachkov et al. 2013) using the default parameters. To extract binding and non-binding motifs, we compared the positions of the motifs obtained with peaks that appeared in two or more replicates as determined by experimental CTCF occupancy analyses (**SM1.11**). To extract motifs occurring in B2_Mm1 retrotransposons, we compared the positions of motifs obtained with the repeat annotation in **SM1.9**. For each category (All binding, No B2Mm1 binding, All not binding, No B2Mm1 Not binding), we counted the occurrence of all possible 64 motif triplet. To normalize the triplets counts, we computed the z score per each category where the mean is the mean of counts of all 64 motif triplets within each category. The results were plotted using the heatmap.2 function of the R gplots package.

SM 5.9 – CTCF turnover analysis

We tested whether B2_mm1 mediated CTCF binding gains are associated with increased turnover of CTCF. Using Enredo-Pecan-Ortheus whole genome alignments (**SM1.13**), we dived *Mus caroli* specific CTCF binding gains into those in the B2Mm1 “binding cluster” extracted from the neighbour-joining classification (**SM5.5**, **Fig. 4D**), those not in a B2Mm1 and also surveyed caroli specific CTCF binding losses as a control. For each of these three categories we found the closest non-overlapping CTCF binding event. The closest binding events were divided into: a “caroli specific gain” if they did not overlap within whole genome alignments with CTCF binding in any other rodent, a “caroli specific loss” if they were present in all other rodents but absent from caroli, and “shared” if they overlapped with at least one other rodent. Finally, those CTCF binding events that had no neighbouring CTCF binding within 50kb were classified as not having a close CTCF binding site. To test whether B2Mm1 associated CTCF binding gains tend to appear in CTCF rich regions of the genome, we tested if the CTCF binding sites appearing near other CTCF binding events (gains, losses or shared) or in deserts (no CTCF binding) were significantly different between B2Mm1 associated and not-associated species-specific gains using Fisher’s exact test in R.

References

Altschul SF, Gish W, Miller W, Myers EW, Lipman DJ. 1990. Basic local alignment search tool. *J Mol Biol* **215**: 403-410.

Ashkenazy H, Penn O, Doron-Faigenboim A, Cohen O, Cannarozzi G, Zomer O, Pupko T. 2012. FastML: a web server for probabilistic reconstruction of ancestral sequences. *Nucleic Acids Res* **40**: W580-584.

Bailey TL, Johnson J, Grant CE, Noble WS. 2015. The MEME Suite. *Nucleic Acids Res* **43**: W39-49.

Bouckaert R, Heled J, Kuhnert D, Vaughan T, Wu CH, Xie D, Suchard MA, Rambaut A, Drummond AJ. 2014. BEAST 2: a software platform for Bayesian evolutionary analysis. *PLoS Comput Biol* **10**: e1003537.

Carbone L, Vessere GM, ten Hallers BF, Zhu B, Osoegawa K, Mootnick A, Kofler A, Weinberg J, Rogers J, Humphray S et al. 2006. A high-resolution map of synteny disruptions in gibbon and human genomes. *PLoS Genet* **2**: e223.

Cunningham F, Amode MR, Barrell D, Beal K, Billis K, Brent S, Carvalho-Silva D, Clapham P, Coates G, Fitzgerald S et al. 2015. Ensembl 2015. *Nucleic Acids Res* **43**: D662-669.

Emes RD, Riley MC, Laukaitis CM, Goodstadt L, Karn RC, Ponting CP. 2004. Comparative evolutionary genomics of androgen-binding protein genes. *Genome Res* **14**: 1516-1529.

Finn RD, Clements J, Eddy SR. 2011. HMMER web server: interactive sequence similarity searching. *Nucleic Acids Res* **39**: W29-37.

Gnerre S, Maccallum I, Przybylski D, Ribeiro FJ, Burton JN, Walker BJ, Sharpe T, Hall G, Shea TP, Sykes S et al. 2011. High-quality draft assemblies of mammalian genomes from massively parallel sequence data. *Proc Natl Acad Sci U S A* **108**: 1513-1518.

Haas BJ, Papanicolaou A, Yassour M, Grabherr M, Blood PD, Bowden J, Couger MB, Eccles D, Li B, Lieber M et al. 2013. De novo transcript sequence reconstruction from RNA-seq using the Trinity platform for reference generation and analysis. *Nat Protoc* **8**: 1494-1512.

Harrow J, Frankish A, Gonzalez JM, Tapanari E, Diekhans M, Kokocinski F, Aken BL, Barrell D, Zadissa A, Searle S et al. 2012. GENCODE: the reference human genome annotation for The ENCODE Project. *Genome Res* **22**: 1760-1774.

Herrero J, Muffato M, Beal K, Fitzgerald S, Gordon L, Pignatelli M, Vilella AJ, Searle SM, Amode R, Brent S et al. 2016. Ensembl comparative genomics resources. *Database (Oxford)* **2016**.

Hubbard TJ, Aken BL, Ayling S, Ballester B, Beal K, Bragin E, Brent S, Chen Y, Clapham P, Clarke L et al. 2009. Ensembl 2009. *Nucleic Acids Res* **37**: D690-697.

Jacobs L, Flynn L. 2005. Of mice… again: the Siwalik rodent record, murine distribution, and molecular clocks. *Interpreting the Past: Essays on Human, Primate, and Mammal Evolution in Honor of David Pilbeam*: 63-80.

Katoh K, Standley DM. 2013. MAFFT multiple sequence alignment software version 7: improvements in performance and usability. *Mol Biol Evol* **30**: 772-780.

Keane TM, Goodstadt L, Danecek P, White MA, Wong K, Yalcin B, Heger A, Agam A, Slater G, Goodson M et al. 2011. Mouse genomic variation and its effect on phenotypes and gene regulation. *Nature* **477**: 289-294.

Kolmogorov M, Armstrong J, Raney BJ, Streeter I, Dunn M, Yang F, Odom D, Flicek P, Keane T, Thybert D et al. 2016. Chromosome assembly of large and complex genomes using multiple references. *bioRxiv* doi:10.1101/088435.

Konig S, Romoth LW, Gerischer L, Stanke M. 2016. Simultaneous gene finding in multiple genomes. *Bioinformatics* **32**: 3388-3395.

Kurtz S, Phillippy A, Delcher AL, Smoot M, Shumway M, Antonescu C, Salzberg SL. 2004. Versatile and open software for comparing large genomes. *Genome Biol* **5**: R12.

Lai W, Choudhary V, Park PJ. 2008. CGHweb: a tool for comparing DNA copy number segmentations from multiple algorithms. *Bioinformatics* **24**: 1014-1015.

Langmead B, Salzberg SL. 2012. Fast gapped-read alignment with Bowtie 2. *Nat Methods* **9**: 357-359.

Laukaitis CM, Heger A, Blakley TD, Munclinger P, Ponting CP, Karn RC. 2008. Rapid bursts of androgen-binding protein (Abp) gene duplication occurred independently in diverse mammals. *BMC Evol Biol* **8**: 46.

Letunic I, Bork P. 2016. Interactive tree of life (iTOL) v3: an online tool for the display and annotation of phylogenetic and other trees. *Nucleic Acids Res* **44**: W242-245.

Li H, Durbin R. 2010. Fast and accurate long-read alignment with Burrows-Wheeler transform. *Bioinformatics* **26**: 589-595.

Lopez Antonanzas R. 2009. First Potwarmus from the Miocene of Saudi Arabia and the early phylogeny of murines (Rodentia: Muroidea). *Zool J Linn Soc* **156**: 664-679.

McKenna A, Hanna M, Banks E, Sivachenko A, Cibulskis K, Kernytsky A, Garimella K, Altshuler D, Gabriel S, Daly M et al. 2010. The Genome Analysis Toolkit: a MapReduce framework for analyzing next-generation DNA sequencing data. *Genome Res* **20**: 1297-1303.

Navarro FC, Galante PA. 2013. RCPedia: a database of retrocopied genes. *Bioinformatics* **29**: 1235-1237.

Pachkov M, Balwierz PJ, Arnold P, Ozonov E, van Nimwegen E. 2013. SwissRegulon, a database of genome-wide annotations of regulatory sites: recent updates. *Nucleic Acids Res* **41**: D214-220.

Park NS, L.; Gu, Y.; Keane, T.; Swerdlow, H.; Quail, M. 2013. An improved approach to mate-paired library preparation for Illumina sequencing. *Methods in Next Generation Sequencing* **1**.

Paten B, Earl D, Nguyen N, Diekhans M, Zerbino D, Haussler D. 2011. Cactus: Algorithms for genome multiple sequence alignment. *Genome Res* **21**: 1512-1528.

Paten B, Herrero J, Beal K, Fitzgerald S, Birney E. 2008a. Enredo and Pecan: genome-wide mammalian consistency-based multiple alignment with paralogs. *Genome Res* **18**: 1814-1828.

Paten B, Herrero J, Fitzgerald S, Beal K, Flicek P, Holmes I, Birney E. 2008b. Genome-wide nucleotide-level mammalian ancestor reconstruction. *Genome Res* **18**: 1829-1843.

Pei B, Sisu C, Frankish A, Howald C, Habegger L, Mu XJ, Harte R, Balasubramanian S, Tanzer A, Diekhans M et al. 2012. The GENCODE pseudogene resource. *Genome Biol* **13**: R51.

Rice P, Longden I, Bleasby A. 2000. EMBOSS: the European Molecular Biology Open Software Suite. *Trends Genet* **16**: 276-277.

Rodriguez JM, Maietta P, Ezkurdia I, Pietrelli A, Wesselink JJ, Lopez G, Valencia A, Tress ML. 2013. APPRIS: annotation of principal and alternative splice isoforms. *Nucleic Acids Res* **41**: D110-117.

Schmidt D, Schwalie PC, Wilson MD, Ballester B, Goncalves A, Kutter C, Brown GD, Marshall A, Flicek P, Odom DT. 2012. Waves of retrotransposon expansion remodel genome organization and CTCF binding in multiple mammalian lineages. *Cell* **148**: 335-348.

Schmidt D, Wilson MD, Spyrou C, Brown GD, Hadfield J, Odom DT. 2009. ChIP-seq: using high-throughput sequencing to discover protein-DNA interactions. *Methods* **48**: 240-248.

Schwalie PC, Ward MC, Cain CE, Faure AJ, Gilad Y, Odom DT, Flicek P. 2013. Co-binding by YY1 identifies the transcriptionally active, highly conserved set of CTCF-bound regions in primate genomes. *Genome Biol* **14**: R148.

Sievers F, Wilm A, Dineen D, Gibson TJ, Karplus K, Li W, Lopez R, McWilliam H, Remmert M, Soding J et al. 2011. Fast, scalable generation of high-quality protein multiple sequence alignments using Clustal Omega. *Mol Syst Biol* **7**: 539.

Simao FA, Waterhouse RM, Ioannidis P, Kriventseva EV, Zdobnov EM. 2015. BUSCO: assessing genome assembly and annotation completeness with single-copy orthologs. *Bioinformatics* **31**: 3210-3212.

Sisu C, Pei B, Leng J, Frankish A, Zhang Y, Balasubramanian S, Harte R, Wang D, Rutenberg-Schoenberg M, Clark W et al. 2014. Comparative analysis of pseudogenes across three phyla. *Proc Natl Acad Sci U S A* **111**: 13361-13366.

Slater GS, Birney E. 2005. Automated generation of heuristics for biological sequence comparison. *BMC Bioinformatics* **6**: 31.

Stanke M, Diekhans M, Baertsch R, Haussler D. 2008. Using native and syntenically mapped cDNA alignments to improve de novo gene finding. *Bioinformatics* **24**: 637-644.

Stanke M, Tzvetkova A, Morgenstern B. 2006. AUGUSTUS at EGASP: using EST, protein and genomic alignments for improved gene prediction in the human genome. *Genome Biol* **7** **Suppl 1**: S11 11-18.

Su AI, Wiltshire T, Batalov S, Lapp H, Ching KA, Block D, Zhang J, Soden R, Hayakawa M, Kreiman G et al. 2004. A gene atlas of the mouse and human protein-encoding transcriptomes. *Proc Natl Acad Sci U S A* **101**: 6062-6067.

Ward BJ, van Oosterhout C. 2016. HYBRIDCHECK: software for the rapid detection, visualization and dating of recombinant regions in genome sequence data. *Mol Ecol Resour* **16**: 534-539.

Yang F, Graphodatsky AS. 2017. Animal Probes and ZOO-FISH. In *Fluorescence In Situ Hybridization (FISH): Application Guide*, doi:10.1007/978-3-662-52959-1_42 (ed. T Liehr), pp. 395-415. Springer Berlin Heidelberg, Berlin, Heidelberg.

Yue F Cheng Y Breschi A Vierstra J Wu W Ryba T Sandstrom R Ma Z Davis C Pope BD et al. 2014. A comparative encyclopedia of DNA elements in the mouse genome. *Nature* **515**: 355-364.

Zhang Y, Liu T, Meyer CA, Eeckhoute J, Johnson DS, Bernstein BE, Nusbaum C, Myers RM, Brown M, Li W et al. 2008. Model-based analysis of ChIP-Seq (MACS). *Genome Biol* **9**: R137.

Zhang Z, Carriero N, Zheng D, Karro J, Harrison PM, Gerstein M. 2006. PseudoPipe: an automated pseudogene identification pipeline. *Bioinformatics* **22**: 1437-1439.
